# Inhibition of saccade initiation improves saccade accuracy: The role of local and remote visual distractors in the control of saccadic eye movements

**DOI:** 10.1167/jov.21.3.17

**Published:** 2021-03-17

**Authors:** Eugene McSorley, Alice G. Cruickshank, Rachel McCloy

**Affiliations:** 1 School of Psychology and Clinical Language Sciences, University of Reading, Berkshire, UK; 2 School of Psychology and Clinical Language Sciences, University of Reading, Berkshire, UK; 3 School of Psychology and Clinical Language Sciences, University of Reading, Berkshire, UK

**Keywords:** direction, distance, virtual reality, cue combination, Bayesian

## Abstract

When a distractor appears close to the target location, saccades are less accurate. However, the presence of a further distractor, remote from those stimuli, increases the saccade response latency and improves accuracy. Explanations for this are either that the second, remote distractor impacts directly on target selection processes or that the remote distractor merely impairs the ability to initiate a saccade and changes the time at which unaffected target selection processes are accessed. In order to tease these two explanations apart, here we examine the relationship between latency and accuracy of saccades to a target and close distractor pair while a remote distractor appears at variable distance. Accuracy improvements are found to follow a similar pattern, regardless of the presence of the remote distractor, which suggests that the effect of the remote distractor is not the result of a direct impact on the target selection process. Our findings support the proposal that a remote distractor impairs the ability to initiate a saccade, meaning the competition between target and close distractor is accessed at a later time, thus resulting in more accurate saccades.

## Introduction

Natural limitations of the visual system mean we must move our eyes to gather visual information about our environment (Findlay & Gilchrist, 2003; Land & Tatler, 2009). These environments, such as the natural world, artificial visual displays, and so on, contain multiple visual stimuli from which we select the target for our eye movements. Targets are usually self-evident, in that we know what it is we are looking for. They will often relate to the current task with which we are occupied. Selection of the next saccadic eye movement is suggested to be the outcome of a competition between different potential visual targets (McPeek, 2006; Purcell et al., 2010; Schall, 2015; Trappenberg, Dorris, Munoz, & Klein, 2001), resulting in the generation of a saliency and/or priority map for target selection (Awh, Belopolsky, & Theeuwes, 2012; Fecteau & Munoz, 2006; Itti & Koch, 2000; McPeek, Skavenski, & Nakayama, 2000). The development of this target-selection competition is reflected in modulation of saccade response time and landing position when other, distracting stimuli and perhaps fixation must be inhibited, that is, when and where the eyes are directed reflects the state of the underlying target-selection competition at the time of saccade initiation, perhaps coupled with time necessary to disengage from fixation (Findlay, 1982, Findlay & Walker, 1999; Glimcher & Sparks, 1993; Kopecz, 2003; Ludwig & Gilchrist, 2003; McPeek, 2006; McPeek, Han, & Keller, 2003; McSorley & Findlay, 2003; McSorley, Haggard, & Walker, 2006; Meeter, van der Stigchel, & Theeuwes, 2010; van Opstal & van Gisbergen, 1989). It has been proposed that facilitation and inhibition of the saccadic response comes from a variety of sources, including attentional processes posited in Lateral Intraparietal Cortex (LIP) (Bisley & Mirpour, 2019) and processes of discrimination and selection of the target, largely situated in Frontal Eye Fields (FEF) (Schall, 2015). This reflects underlying short-range Mexican hat–style excitatory and long-range inhibitory connectivity across multiple visual and saccade-related areas throughout the brain (Cavanaugh, Joiner, & Wurtz, 2012; Fecteau & Munoz, 2006; Fino & Yuste, 2011; Leigh & Zee, 2006; Moschovakis, Scudder, & Highstein, 1996; Munoz & Fecteau, 2002; Munoz & Istvan, 1998; Munoz & Wurtz, 1993; Olivier, Dorris, & Munoz, 1999). It has been suggested that the final response latency of a saccade reveals the time taken for activity induced by target selection processes to overcome any inhibition to reach a threshold level necessary to trigger a movement (Hanes & Schall, 1996; Jantz, Watanabe, Everling, & Munoz, 2013). This also includes a contribution from oculomotor activation associated with programming of saccades in other nontarget directions (Munoz & Istvan, 1998). The time taken for target-related activity to reach threshold and initiate a saccadic movement is also directly related to reducing activation required to maintain fixation (Munoz & Wurtz, 1993), such as that controlling small drift movements and coding small microsaccades serving to hold the eyes steady on an object or area of interest (Hafed, Goffart, & Krauzlis, 2009; Ko, Poletti, & Rucci, 2010).

It has been shown that when a single visual distractor accompanies a visual target, both saccade latency and landing position (or accuracy) are dependent upon their spatial separation. Generally, little effect on saccade latency has been found when a target and distractors are close together (within a sector of about 20–30 degrees of the target), although shortening of latencies has, on occasion, been reported in these circumstances (speeding: Edelman & Xu, 2021; Godijn & Theeuwes, 2002; McSorley, Cruickshank, & Inman, 2009, McSorley, McCloy & Lyne, 2012; Walker, Deubel, Schneider, & Findlay, 1997; slowing: Chou et al., 1999; Sailer et al., 2002; no difference: Bompas & Sumner, 2011; McSorley & Findlay, 2003). Rather, it is both saccade landing position and its trajectory that are most reliably affected. Saccade trajectories have been found to curve toward distractors before finally landing somewhere between a target and distractor (McSorley et al., 2006; Mulckhuyse, Van der Stigchel, & Theeuwes, 2010). The actual trajectory and landing position depends on a number of factors such as separation, saliency, discriminability, the continuous availability of visual information, and saccade latency (Arkesteijn, Donk, Smeets, & Belopolsky, 2020; Arkesteijn, Smeets, Donk, & Belopolsky, 2018; Chou, Sommer, & Schiller, 1999; Coëffé & O'Regan, 1987; Deubel, Wolf, & Hauske, 1984; Findlay, 1981, 1982; Glimcher & Sparks, 1993; Heeman, Theeuwes, & van der Stigchel, 2014; Lee, Rohrer, & Sparks, 1998; McIlwain, 1986, 1991; McSorley, Cruickshank, & Inman, 2009; McSorley & Findlay, 2003; Ottes, van Gisbergen, & Eggermont, 1984, 1985; van Opstal & van Gisbergen, 1989; van der Stigchel, 2010; Walker & McSorley, 2008; Walker et al., 1997). The effect on landing position is known as the global effect or the center of gravity effect (Findlay, 1981; Findlay & Brown, 2006a, 2006b; He & Kowler, 1989). In terms of a priority map for saccade target selection, which reflects contributions from bottom-up stimulus saliency and top-down task demands (such as selection history and experience), a saccade is directed toward the area of maximum activity (e.g., Glimcher & Sparks, 1993; Lee et al., 1998; Munoz & Wurtz, 1995; Port & Wurtz, 2003). Hence, saccade landing position is a function of activity at target and distractor locations at the time the movement is triggered, which results in a saccade being directed toward an “average” site somewhere between the two locations (Findlay, 1982; Godijn & Theeuwes, 2002). The global effect is usually attributed to distributed spatial coding in Superior Colliculus (SC) (Edelman & Keller, 1998; van Opstal & van Gisbergen, 1989). Here the deep and intermediate layers of SC are populated with neurons with large overlapping receptive and movement fields that respond retinotopically to visual space such that areas in close spatial proximity contribute to multiple receptive fields centered on adjoining locations (Phongphanphanee et al., 2014; Vokoun, Huang, Jackson, & Basso, 2014). In this way, potential saccade targets in close proximity elicit activation across overlapping neuronal populations in the deep and intermediate layers of SC so that their receptive/movement fields overlap and receive activation from multiple potential targets, and hence saccades are directed to those that, of course, represent visual locations somewhere between the targets (Findlay & Walker, 1999; van Opstal & van Gisbergen, 1989). This is likely to be a function of an interplay between short-range and long-range connections across SC. However, it has also been suggested that the global effect arises from the weighted average of the entire active population in SC (Lee, Rohrer, & Sparks, 1998; Meeter et al., 2010; Robinson, 1972). In terms of saliency and priority maps for saccade control, it has been hypothesized that the lateral interparietal area, frontal eye fields, and interactions in different layers in SC play a key role in the computation of both of these prior to feeding into a final “winner-take-all” function in the deep layers of SC (Bisley & Mirpour, 2019; Veale, Hafed, & Yoshida, 2017).

When stimuli are separated further, saccade accuracy is not affected, but both the trajectory and latency of the saccade are. Rather than curve toward the distractor, the saccade now deviates away from its location (e.g., McSorley et al., 2012; for reviews, see van der Stigchel, 2010; Walker & McSorley, 2008; White, Theeuwes, & Munoz, 2012). Saccade latency reliably increases, and this is known as the remote distractor effect (Bompas & Sumner, 2009; Born & Kerzel, 2008; Dorris et al., 2007; Griffiths, Whittle, & Buckley, 2006; Honda, 2005; Levy-Schoen, 1969; Ludwig, Gilchrist, & McSorley, 2005a; McSorley, McCloy, & Lyne, 2012; Ross & Ross, 1980; Walker, Fitzgibbon, & Goldberg, 1995; Walker, Kentridge, & Findlay, 1995; Walker et al., 1997; White, Gegenfurtner, & Kerzel, 2006). The remote distractor effect occurs even when higher-level cognitive components involved in target selection and search have been minimized by restricting the target to appear in only a very small number of potential locations (e.g., restricted to appear in a single axis of one visual hemifield: on one side of fixation while the distractor appears in the opposite hemifield), suggesting it is an automatic effect, not subject to any kind of top-down overriding control (Benson, 2008; McSorley et al., 2012; Walker et al., 1995; Walker et al., 2000). The magnitude of the remote distractor effect has also been found to increase as remote distractor distance from fixation gets smaller (McSorley, Cruickshank, & Inman, 2009; McSorley, Haggard, & Walker, 2009; Walker et al., 1997) and has been shown to depend on the distance of the remote distractor from the target both when the target can appear in multiple locations and when it is restricted to a very few locations in a single hemifield (Godijn & Theeuwes, 2002; McSorley, Cruickshank, & Inman, 2009; McSorley, Haggard, & Walker, 2004, 2009; McSorley et al., 2012; Van der Stigchel, Meeter, & Theeuwes, 2007). The remote distractor effect is especially large when it is presented centrally or in the opposite hemifield to the target, with the largest and most consistent effect being found when the target to distractor angular separation is over 150 degrees, what McSorley et al. (2012) termed the opposite target effect. Studies have also shown the remote distractor effect to be temporally dependent on distractor onset, with the largest effect occurring for onsets within a 20-ms window of target onset (Bompas & Sumner, 2009). Lengthening of saccade latency has also been reported for distractor onsets 80 ms before and up to 60 ms after the target, depending on the contrast of the distractor (McSorley, McCloy, & Lyne, 2012; Reingold & Stampe, 2002; Walker, Fitzgibbon, & Goldberg, 1995; White et al., 2006). In a separate but somewhat related research field using the Posner cuing paradigm, it has been reported that cues (potential distractors) produce a speeding or a slowing of saccade response dependent on whether the distractor was shown just 50 ms prior to target onset, thus acting as a cue in this instance, or 200 ms afterward (labeled *inhibition of* *return*), respectively (Fecteau, Bell, & Munoz, 2004; Fecteau & Munoz, 2006; Khan, Heinen, & McPeek, 2010; Khan, Munoz, Takahashi, Blohm, & McPeek, 2016; Klein, 2000; Posner & Cohen, 1984; Posner, Rafal, Choate, & Vaughan, 1985).

Despite each playing a fundamental role in determining the parameters of a saccade, the relationship between the global effect and the remote distractor effect has been little explored. Some evidence shows little relationship between them, suggesting that the control of where and when saccades are directed is somewhat separable. For example, Casteau and Vitu-Thibault (2012) showed that targets accompanied by an ipsilateral distractor on the same axis elicited a range of effects from no effect to a speeding or a slowing of target-directed saccades depending on eccentricity but did show a global effect. McSorley et al. (2009) showed that ipsilateral off-axis distractors showed a global effect in a 20-degree window of the target and lengthened saccade latency at greater separations, and Walker et al. (1997) showed this effect for on-axis targets. However, a basic relationship between saccade latency and accuracy has been documented on numerous occasions with shorter latency saccades tending to land closer to a distractor, exposing the influence of distractor activity (e.g., Chou et al., 1999; Coëffé & O'Regan, 1987; Findlay, 1981). It has been suggested that longer latency saccades land closer to the target because competition between potential target stimuli has further developed, that is, closer-to-target distractor stimuli are more successfully inhibited, target activation is heightened, and thus the location of the target becomes more finely resolved (Ottes et al., 1985). Furthermore, McSorley and Findlay (2003) found that the accuracy of saccades made in response to a target and a single nearby distractor was improved when the number of other distractor stimuli present was increased, which led to a rise in target-directed saccade latency. They suggested this was due to the increased number of distractors present acting as remote distractors, which increased saccade latencies and improved target-directed accuracy. Cruickshank and McSorley (2009) provided further support for this. They report that making the location of potential targets and distractors presented close to them more certain (e.g., by restricting them to appear in one hemifield) led to accuracy improvements even when a further distractor was shown remote from them was presented in the opposite hemifield. Thus, the pattern of saccade accuracy improvement found in the presence of the remote distractor mirrors that found in the natural variation of saccade latencies. For example, if a display showing a target and close distractor on the horizontal meridian at 6 degrees and 3 degrees, respectively, elicited a hypometric saccade with a saccade latency of 280 ms and a 4-degree amplitude, then it would be expected that if a similarly timed saccadic response was made to the same target-distractor configuration when an additional remote distractor was also present, then the same saccade accuracy would be expected. In the latter case, the remote distractor slowed a naturally occurring shorter latency response (e.g., a saccade of 260-ms latency became a 280-ms response when the remote distractor was present) by interrupting fixation disengagement, and the saccade accuracy was unaffected compared with a similarly timed response when no remote distractor was present. Note this suggestion is diametrically opposed to the finding reported by Casteau and Vitu-Thibault (2012), in which the presence of the ipsilateral distractor slowed or showed no effect on (not speeded) saccade latency and induced a global effect. However, in that particular case, the ipsilateral distractor was close to fixation and on the same axis as the target, unlike the effects from the off-axis distractor(s) or contralateral discussed earlier in this paragraph (Cruickshank & McSorley, 2009; McSorley & Findlay, 2003; McSorley et al., 2009). Indeed, Casteau and Vitu-Thibault (2012) also reported that contralateral distractors induced a remote distractor effect with no global effect.

Despite these somewhat mixed findings, it is likely that, as well as there being a natural relationship between saccade latency and accuracy, there is a more direct consequence of remote distractor presence on where target-directed saccades land. Indeed, reports that the remote distractor effect increases with increased distance from a target, despite distance from fixation remaining constant, suggests that there is a more direct interaction between remote distractor and the saccade targeting processes (McSorley et al., 2009; McSorley, et al., 2012; cf. Walker et al., 1997). If this was the case, the suggestion could be that the remote distractor not only impairs fixation disengagement but also impacts directly on target-selection processes taking place between the target and close distractor such that both the latency and accuracy of the saccadic response are affected (a “direct” hypothesis of the process).

Generally speaking, the findings from effects of remote distractors on saccade latency and landing positions can be interpreted in two ways, both of which work within the context of saccades being directed to the highest point of activation on a priority map. One is that there are competitive interactions between a fixate system and a move system. Thus, the increase in target-directed saccade latency is due to the indirect effect of the presence of a remote distractor, which hampers fixation disengagement, thus slowing saccade initiation by increasing activation at fixation (i.e., it does not directly impact on target activation itself) (Casteau & Vitu-Thibault, 2012; Findlay & Walker, 1999). The eye is held steady until activation in the fixation system reduces below a threshold, at which point the eyes move to the highest point of activation on a spatiotopic map coding visual space. The shift in the balance of activation is a reciprocal relationship. In this “indirect” or “fixation gating” model, the remote distractor effect results from enhanced activity in the fixate system. As stated above, the second hypotheses posited to account for remote distractor effect is that it is the result of direct competitive interactions between the target and distractor alone with no involvement of a fixation gating system at all. Underlying the front end of both these explanations for the control of where and when saccades are executed is the same: Eyes are fixated until activity at fixation is reduced below a threshold, at which point the eyes move to the area of highest activation on the neural movement map representing visual space of potential saccade targets. The difference between the two hypotheses is the operation of an indirect extended fixation zone, which acts as a gating system to saccade execution. In the fixation gating hypothesis, target-directed saccade latency is an indirect consequence of activation at fixation, which does not directly impact on target activation. In the second hypothesis, the appearance of a remote distractor impacts directly on target activation via lateral interactions but not indirectly on fixation activation. What slows fixation disengagement here is not an enhanced activation at fixation caused by distractor-to-fixation interactions. Rather, it is the direct interaction between the remote distractor and target that slows saccade threshold triggering (perhaps by the remote distractor activation interfering with target activation reaching a triggering threshold), thus slowing saccade onset.

When considered in terms of the underlying neurophysiology of the saccadic eye movement system, there is evidence that could be taken to support both hypotheses. The indirect fixation gating hypothesis originally equated the fixate system with fixation neurons identified at the rostral pole of SC (a region receiving input from the 2-degree foveal area; Munoz & Wurtz, 1993). However, recent findings have cast doubt on the existence of these fixation neurons per se. Current evidence supports a conception of SC that is more of a continuous map coding for locations in visual space at increasing eccentricities from fixation as the SC is tracked caudally away from the rostral pole (Hafed, Goffart, & Krauzlis, 2009; Ko, Poletti, & Rucci, 2010). Locations in visual space closer to the current point of fixation are coded close to the rostral pole in SC so that it represents more of a continuous visuomotor map of visual space that topographically mirrors visual space. Crucially, neurons coding for extremely small-amplitude microsaccades act in the same manner as those coding for larger amplitudes (Hafed, 2011). Given these findings, it is much more likely that a fixate system exists downstream of SC in the brainstem. Here we find omnipause neurons that discharge tonically during fixation, holding the eyes steady and ceasing to respond, that is, they pause, during saccades. These receive excitation from the rostral pole of SC and less from neurons located more caudally on SC, as distant as those located 10 degrees from fixation (Büttner-Ennever, Horn, Henn, & Cohen, 1999; Everling, Pare, Dorris, & Munoz, 1998; Gandhi & Keller, 1999). This has been referred to as an “extended fixation zone” by Walker et al. (1997) and further developed by Findlay and Walker (1999) so that even peripheral excitation can elicit enhanced activity at fixation and result in slower saccadic responses (i.e., disengagement from fixation). Thus, a slowing in the saccade response to a target onset would be a function of the activation in saccade-related areas throughout the brain caused by target onset and the current activation at fixation, which would slow disengagement (increase time for Omnipause Neurons (OPN) to cease firing). Any activation caused by a further visual, remote distractor may impact on fixation activation either through interactions with the target itself or via the “extended fixation zone” (if applicable). Interactions with the target itself would be a direct result of excitatory or inhibitory connections between the target and remote distractor in a variety of areas involved in saccade control throughout the eye movement system.

The second more “direct” conception of target-directed saccade control posits “where” the eyes are directed and also controls “when” the eyes are moved. This results from competition in the SC via horizontal connections within and between colliculi, where, in a Mexican hat–style connectivity, there are short-range excitatory interactions and long-range inhibitory interconnections between areas on the SC neural map of visual space (Phongphanphanee et al., 2014; Vokoun, Huang, Jackson, & Basso, 2014). Here intercollicular connections would be inhibitory (except at the rostral pole). This interplay of remote distractor and target onset would evoke a competitive process that, via long-range interconnections in SC, show neural activation at a single final saccade target site when stimuli are of a sufficiently large separation. However, if a distractor is located close to the target, then their activation pools together via horizontal excitatory connections and leads to a site of maximal activation between the target and distractor, resulting in saccades that land between their actual locations (averaging saccades). Here, then, the interstimulus distance determines both the where and when of the saccade (i.e., both its metrics and dynamics).

However, it has not been shown whether the latency of a saccade depends solely on an indirect fixation gating system or relies on more direct target-to-distractor interactions. This may be due to the difficulty in isolating the effect of distractor presence on target activation as distinguished from fixation activation both via horizontal connections between sites in SC and across colliculi. The variety of effects on saccade landing position and latency are likely due to the imbalance in activity at those sites at the time of saccade execution, as well as during the saccade itself (see Buonocore et al., 2020). It would be predicted, then, that a distractor shown close to fixation should slow saccade latency, whereas as it approaches the target, latency should speed up. Indeed, Casteau and Vitu-Thibault (2012) showed in three experiments that distractor distance from fixation was the primary determinant of saccade latency. There was little to no effect of distractor distance to target. They report three experiments, in which distractor distance from target varied across and within the same visual field (i.e., both ipsilateral and contralateral to target) with distractor and targets shown at very small eccentricities. In these experiments, target identity had to be indicated by button press, and the fixation period prior to stimulus onset was exceedingly short. In all experiments, they found that as distance to target increased, there was no change in saccade latency, but as distance to fixation decreased, latencies increased. This pattern of results is inconsistent with a purely lateral interaction account of remote distractor effect and favors an indirect fixation gating system account. It is also worth noting that both Walker et al. (1997) and Casteau and Vitu-Thibault (2012) also showed that the extent of the remote distractor effect could be tied not just to the distance of the distractor from fixation but also to the ratio of distractor distance from fixation to target distance from fixation. Furthermore, Casteau and Vitu-Thibault (2012) also showed no global effect for distractors shown contralateral to the target or for those ipsilateral with the target but close to fixation. The global effect was only shown when the distractor was shown 2 or 3 degrees from fixation across a range of target distances despite these responses being a function of longer, not shorter, latencies than shown at closer distractor locations. This pattern of results is not predicted by a more direct interaction model. This would predict both a global effect and a speeding (a reduction) in saccade latency, not the slowing, as was found. As an increase in saccade latency was reported, the prediction would be that interactions between target and distractor should be greatly resolved around the target and thus show less of a global effect. Overall, behavioral data show that saccade latency is a function of relative eccentricity of target and distractor from fixation, not a direct function of distractor distance from the target. Thus, the latency of the saccade toward an eccentric target seems to be under the control of a fixation gating system, and while lateral interactions play an important role, they are not solely responsible for this.

In a pointed attempt to examine the indirect and direct hypotheses of target and distractor interactions on saccade control (i.e., the latency and landing position of the response), McSorley and Cruickshank (2010) recorded saccade responses made to a target that may be accompanied by a close distractor that was always presented in one hemifield. They also included trials in which a remote distractor was presented in the opposite hemifield. Supporting the indirect hypothesis, they reported similar improvements in saccade accuracy as a function of increases in saccade latency whether the remote distractor was present or not (i.e., the slopes and intercepts that described this relationship were not significantly different). In support of the general behavioral findings of remote distractor presence on saccade control (e.g., that reported by Casteau & Vitu-Thibault, 2012), this suggests that the remote distractor may act by impairing disengagement from fixation only and that it did not directly affect the target selection competition between target and close distractor.

However, there are a number of reasons to be hesitant in interpreting these results as evidence supporting the indirect hypothesis. First, it is possible that the position of the remote distractor used by McSorley and Cruickshank (2010) was simply too far from the target and close distractor locations to impact directly on their competition. Perhaps the remote distractor impaired disengagement from fixation because it was closer to fixation compared to the target and its close distractor. It was shown in the opposite hemifield from the target–close distractor after all. Second, in order to generate a wide range of saccade latencies, McSorley and Cruickshank (2010) manipulated events at fixation (a gap/overlap manipulation). However, given that the effect of the remote distractor is suggested to result in a slowing of disengagement from fixation, it is unclear what effect manipulating events at fixation may have on this process. Similarly, Casteau and Vitu-Thibault (2012) employ extremely short fixation periods and a target identity discrimination task that may also have introduced undesired effects on saccade control outside those involved in the remote distractor effect.

**Figure 1.**
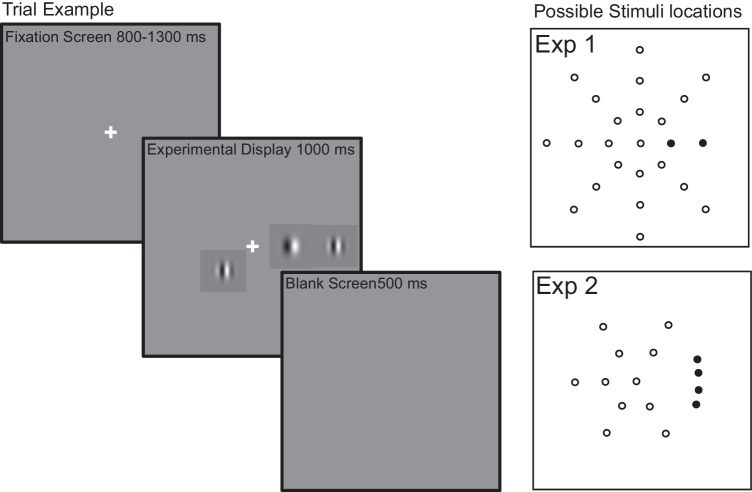
An example trial run is shown on the left and possible target and distractor locations for Experiments 1 and 2 are on the right. Trials start with a fixation cross, shown for between 800 and 1,300 ms. This disappears and simultaneously the experimental stimuli are shown. The target is a low spatial frequency Gabor patch. This can be shown alone or coupled with a close distractor and, very often, a single remote distractor (high spatial frequency Gabor patches). On any trial, the target and close distractor would always be present. A single remote distractor could also be present. The right-hand displays illustrate the possible target and distractor positions for each experiment. Target and close distractor positions are shown as filled circles. That is, in Experiment 1, the target appeared on the horizontal axis at 3 or 6 degrees from fixation while the close distractor appeared at 6 or 3 degrees; in Experiment 2, the target appeared on the 10° or 20° axis above or below the horizontal meridian while the close distractor appeared in the mirror position (if target was shown offset above by 5°, then the close distractor was offset below by the same deviation). The remote distractor, when shown, could appear in any of the locations marked here as unfilled circles (see Method for location information).

In order to address these issues, we report two experiments in which observers were required to saccade to a target in the presence of a close distractor. These stimuli were often accompanied by a further distractor at various, remote locations (see Figure 1). Fixation was removed with the onset of stimuli, thereby making events at fixation equivalent in all trials. As previously stated, saccade extent is known to be a function of the activity of target and close distractor locations (the global effect), and we therefore examined the relationship between saccade accuracy and latency for each remote distractor position by separately examining saccade amplitudes or saccade direction for short and longer latency saccades for trials on which the close distractor was and was not present. Any change in this relationship would indicate an impact of the presence of the remote distractor on the target selection competition between the target and close distractor, supporting the direct hypothesis. If the relationship remains unaffected by the distance of the remote distractor from the target, this suggests an impact of the presence of the remote distractor on fixation-disengagement only, supporting the indirect hypothesis. In both cases, the accuracy improvement results from accessing the competition between the target and close distractor at a more developed stage, but the development underlying them is very different.

## Method

### Observers

Seven different naive observers participated in each experiment, five female and two male in each. Observers ranged in age from 19 to 21. All observers had normal or corrected-to-normal vision. Ethical approval from School of Psychology, University of Reading was obtained for this study, and all participants gave their informed consent prior to inclusion.

### Apparatus and materials

Stimuli were vertically oriented Gabor patches with a spatial frequency of two (target) or four (both close distractor and remote distractor) cycles per degree (cpd), with a standard deviation of 0.3° and a contrast of 90%. All stimuli were presented on a gray background, with a mean luminance of 23 cd/m^2^. Eye movements were recorded using a head-mounted, video-based eye-tracker with a sampling rate of 500 Hz (Eyelink II; SR Research, Ontario, Canada), recording monocularly from observers’ right eyes. Stimuli were presented in grayscale on a 21-in. color monitor with a refresh rate of 75 Hz (DiamondPro; Sony, Weybridge, UK) in sequences developed using Experiment Builder (SR Research). Head movements were constrained with a chinrest, which held participants so their eyes were in line with the horizontal meridian of the screen, at a viewing distance of 1 m. The eye-tracker was calibrated using a standard 9-point grid, carried out at the beginning of the experiment and after any breaks where the observer removed their head from the rest or removed the eye-tracker. Calibration was only accepted once there was an overall difference of less than 0.5° between the initial calibration and a validation retest: In the event of a failure to validate, calibration was repeated.

## Experiment 1

### Design

**Figure 2.**
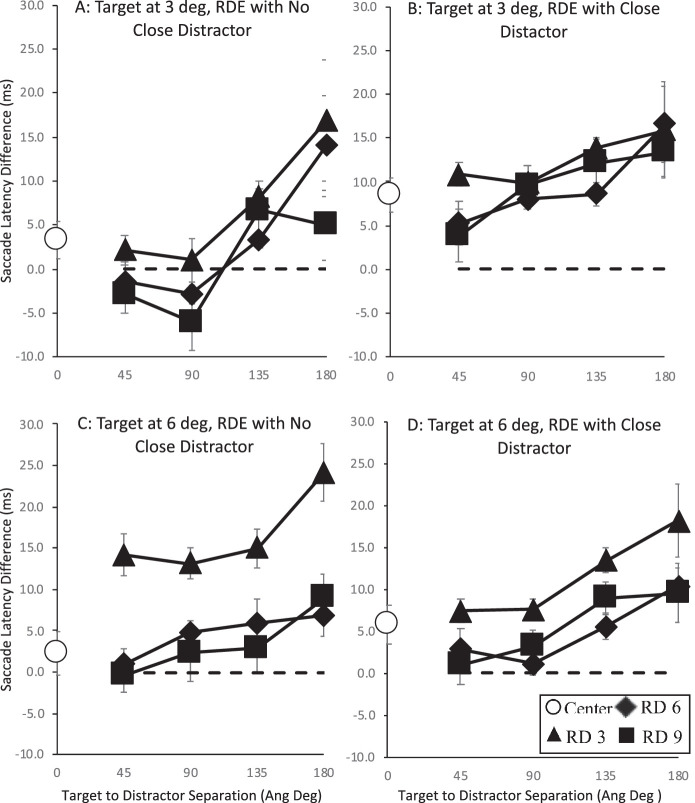
The remote distractor effect as a function of target distance, close distractor presence, and the remote distractor distance from fixation and target. Results when the target location was at 3 degrees of visual angle from fixation are shown in the upper row and from 6 degrees of visual angle in the lower row. The effect of the remote distractor is shown for distances from fixation (3, 6, and 9 degrees of visual angle, labeled RD 3, RD 6, and RD 9) and for each remote distractor distance from the target (45, 90, 135, and 180 angular degrees). Error bars are within-subject error bars (Cousineau, 2005).

In Experiment 1, stimuli were presented on the horizontal meridian at near (3 degree from fixation) or far (6 degree from fixation) locations (see Figure 2 for stimuli layouts for both experiments). A target stimulus was always present. When a close distractor was present, it was in the same hemifield as the target, at the nontarget location (i.e., 6 degree if the target was at 3 degree and vice versa). In Experiment 2, stimuli were presented 6 degrees of visual angle from fixation. A target and close distractor were always present in one of four possible locations: on the 10° or 20° axis above or below the horizontal axis. Target and close distractor were always presented at the same angular offset (e.g., if the target appeared at 10° axis rotation above the horizontal axis, the close distractor would appear at 10° rotation below).

A distractor more remote from the target/close distractor complex could also be present. For Experiment 1, this appeared in a nontarget location 3, 6, or 9 degrees of visual angle from fixation and at an angular deviation of 45°, 90°, 135°, or 180° from the horizontal meridian on which the target lay (see Figure 1). For Experiment 2, the remote distractor appeared in a nontarget location 3 or 6 degrees of visual angle from fixation and at an angular deviation of 60°, 120°, or 180° from the horizontal meridian on which the target lay (see Figure 2). The remote distractor could also appear at the center, replacing the fixation marker (please note that the remote distractor was always further away from the target than the close distractor in both experiments, except for one condition in Experiment 1 when the remote distractor is shown 3 degrees from fixation, 45 angular degrees from the horizontal meridian). For Experiment 1, this gave 2 target locations, 2 close distractor locations, and 22 remote distractor locations. For Experiment 2, this gave 4 target locations, 4 close distractor locations, and 11 remote distractor locations. In both experiments, a baseline condition was also shown in which the target was presented by itself. This allowed a measure of a change in saccade accuracy to be determined. There were 10 trials per condition: Each observer carried out 1,800 trials, 900 leftward and 900 rightward, for both experiments. Observers made saccades to targets shown on the left and on the right in separate sessions.

### Procedure

Observers were shown examples of target and distractor stimuli. Following this, an introductory block of up to 20 trials was presented to familiarize observers to the timing and spatial configuration of the experimental trials. Observers were instructed to move their eyes “as quickly and accurately as possible” to the target Gabor patch, ignoring distractors. Trials began with a central fixation cross (+) subtending 0.5 degrees of visual angle, presented for a varying duration between 800 and 1,300 ms. The fixation cross disappeared at the onset of stimuli, which were displayed for 1 s. This was followed by a blank screen, for 500 ms, and then the reappearance of the fixation cross for the next trial. Once the observer refixated within a 1-degree area, centered on the central cross, the next trial commenced.

### Data analysis

A parser, integral to the eye-tracking software, was used to identify saccade start and endpoints using a 22°/s velocity and 8,000°/s^2^ acceleration criteria (SR Research). Further analysis was undertaken using in-house software developed in MATLAB (MathWorks, Natick, MA). Saccade amplitude, latency, and overall direction were derived from the eye movement records for the first saccade in each trial. Amplitude was defined as the horizontal component of the distance between eye start and endpoint (in degrees of visual angle). Saccade latency is the interval between the onset of the target and the initiation of the saccade (in milliseconds). Direction was defined as the angular deviation of saccade direction (degrees) taken from the initial fixation position to final endpoint, in polar coordinates. Saccades were excluded from further analysis if saccade amplitude was less than 1 degree (Experiment 1: 16%; Experiment 2: 14%) or a blink occurred during the saccade (Experiment 1: 2.5%; Experiment 2: 0.5%).

Data were collapsed across target side sessions (left and rightward saccades) and distance of the remote distractor from target location. In Experiment 1, this is a simple up-and-down operation. Remote distractors at 45°, 90°, and 135° clockwise and counterclockwise from the target are collapsed. This gives 40 trials for these conditions and 20 trials for the 180° position. For Experiment 2, the distance of the clockwise and counterclockwise remote distractors from the target changed depending on its position—for example, if the target is offset upward by 10° and then the counterclockwise remote distractors are 50°, 110°, and 170° from the target, while those clockwise of the target location are 70°, 130°, and 170° from the target. This opposite pattern is found when the target is offset downward, and thus we collapsed data across corresponding target and remote distractor distance (e.g., target 10° up with remote distractor at 60° counterclockwise was collapsed with target 10° down and remote distractor at 60° clockwise; i.e., in both cases, remote distractor was 50° from target). The same operation was carried out for the target offset by 20°, resulting in remote distractor to target distances of 40°, 80°, 100°, 140°, and 160°.

## Results

See Supplementary Materials for tables detailing baseline saccade latencies and amplitudes or directions for all experimental conditions.

### On-axis target locations of 3 and 6 degrees

#### Median saccade latencies

In order to address whether a remote distractor impacts on the accuracy of saccade targeting indirectly by impacting on the latency of the saccade or whether it also has a more direct role to play in saccade accuracy, we first need to establish whether the remote distractor impacted on saccade latency. Given previous work, we would expect to see that the presence of a remote distractor will slow saccade latencies. In order to analyze the dependence of saccade latency on remote distractor presence and position, the following strategy was designed.

The effect of remote distractor presence on median saccade latency was examined for target location both when the close distractor was present and, again separately, when it was not. The presence of remote distractor was not fully factorial, with neither “no remote distractor present” nor “remote distractor present at center” changing as function of remote distractor distance from fixation or remote distractor distance from target. Due to this, the decision was made to examine the no remote distractor present and remote distractor present at center condition separately from the analysis of the effect of remote distractor distance from fixation and target.

#### Effect of remote distractor presence on median saccade latencies

Separate two-way analyses of variance (ANOVAs) were performed depending on close distractor presence with target location (3 degrees and 6 degrees) and remote distractor presence (no remote distractor, remote distractor at center, and a further grouped condition of remote distractor in an “other” position) as factors. In both cases, this showed a main effect of close distractor presence (close distractor not present, *F*(2, 12) = 5.261, *MSE* = 23.992, *p* = 0.023, η_p_^2^ = .467; close distractor present, *F*(2, 12) = 11.897, *MSE* = 27.5, *p* = 0.001, η_p_^2^ = .665) but no effect of target location or interaction (*F*s < 1). Follow-up contrasts of center versus no remote distractor and other versus no remote distractor showed differences in the effect of remote distractor location on saccade latency depending on close distractor presence. When the close distractor was not present, the saccade response slowed only when the remote distractor was shown in the other location (other (*M* = 198 ms, *SE* = 6) vs. none (*M* = 192 ms, *SE* = 6), *F*(1, 6) = 8.173, *MSE* = 61.667, *p* = 0.029, η_p_^2^ = .577), whereas when a close distractor was present, then slower responses were found both when the remote distractor was shown in the center and other location (center (*M* = 193 ms, *SE* = 5) vs. none (*M* = 186 ms, *SE* = 6), *F*(1, 6) = 10.486, *MSE* = 68.119, *p* = 0.018, η_p_^2^ = .636; other (*M* = 195 ms, *SE* = 5) vs. none, *F*(1, 6) = 19.601, *MSE* = 19.601, *p* = 0.004, η_p_^2^ = .766). This pattern of analysis outcomes shows evidence for a remote distractor effect with saccade latencies being generally longer when a remote distractor was present.

#### Effect of remote distractor distance from fixation and target on median saccade latencies

A second series of analyses were then carried out to examine the dependency for the remote distractor effect on target location, remote distractor distance from fixation, and remote distractor distance from the target. To do this, we unpacked the “other” remote distractor proxy variable, with three levels across a distance of 3, 6, and 9 degrees of visual angle from fixation and four levels of 45, 90, 135, and 180 angular degrees from the target position. Thus, three-way ANOVAs were performed across the factors of target location and remote distractor distance from fixation and distance from target separately depending on close distractor presence. For each participant, each trial for those distance conditions was subtracted from the median saccade latency recorded for the equivalent stimulus condition when no remote distractor was present (e.g., for each participant, their median saccade latency elicited when the target was shown at 3 degrees and the close distractor was present at 6 degrees was subtracted from the saccade latency for each trial when the same target and close distractor were shown with the addition of the remote distractor). The average for each remote distractor distance condition was then computed. The resulting average remote distractor effect is shown in Figure 2 by remote distractor distance from fixation (3 to 9 degrees of visual angle) and target (45 to 180 angular degrees). The remote distractor effect elicited by the central remote distractor is also plotted on the figure as a filled white circle on the ordinate. Error bars are within-subjects error bars (Cousineau, 2005).

There was found to be no main effect of target location and no interactions, but there were main effects of remote distractor distance from fixation and distance from target (close distractor not present: distance from fixation, *F*(2, 12) = 23.079, *MSE* = 65.121, *p* < 0.001, η_p_^2^ = .794; distance from target, *F*(3, 18) = 10.079, *MSE* = 105.735, *p* < 0.001, η_p_^2^ = .627; all other *p*s > 0.083; close distractor present: distance from fixation, *F*(2, 12) = 10.05, *MSE* = 39.682, *p* < 0.003, η_p_^2^ = .626; distance from target, *F*(3, 18) = 3.658, *MSE* = 180.88, *p* < 0.032, η_p_^2^ = .379; all other *p*s > 0.365). Further contrasts examining the main effects show similar patterns regardless of close distractor presence. The effect of remote distractor distance from fixation was found to be largest when it was presented close to fixation but reduced to a similar level at greater distances (close distractor not present: 3 (*M* = 204 ms, *SE* = 6) vs. 6 (*M* = 196 ms, *SE* = 6) degrees of visual angle, *F*(1, 6) = 44.531, *MSE* = 19.674, *p* < 0.001; 6 vs. 9 (*M* = 194 ms, *SE* = 6) degrees of visual angle, *F*(1, 6) = 2.385, *MSE* = 19. 862, *p* = 0.173; close distractor present: 3 (*M* = 198 ms, *SE* = 6) vs. 6 (*M* = 193 ms, *SE* = 5) degrees of visual angle, *F*(1, 6) = 17.593, *MSE* = 18.499, *p* = 0.006, η_p_^2^ = .746; 6 vs. 9 (*M* = 194 ms, *SE* = 6) degrees of visual angle, *F*(1, 6) < 1). On the other hand, the effect of remote distractor distance from the target was found to increase as its distance from the target increased, with the largest remote distractor effect found at the furthest distance from the target. This pattern was shown most clearly when the close distractor was not present but was also shown when it was present (close distractor not present: 90 (*M* = 194 ms, *SE* = 6) vs. 45 (*M* = 194 ms, *SE* = 6) angular degrees, *F*(1, 6) < 1; 135 (*M* = 199 ms, *SE* = 6) vs. 90 angular degrees, *F*(1, 6) = 13.554, *MSE* = 25.09, *p* = 0.01, η_p_^2^ = .541; 135 vs. 180 (*M* = 205 ms, *SE* = 7) angular degrees, *F*(1, 6) = 7.08, *MSE* = 63.5, *p* = 0.037, η_p_^2^ = .541; close distractor present: 90 (*M* = 192 ms, *SE* = 5) vs. 45 (*M* = 191 ms, *SE* = 5) angular degrees, *F*(1, 6) = 5.019, *MSE* = 5.138, *p* = 0.066, η_p_^2^ = .455; 135 (*M* = 196 ms, *SE* = 5) vs. 90 angular degrees, *F*(1, 6) = 6.583, *MSE* = 31.638, *p* = 0.043, η_p_^2^ = .523; 135 vs. 180 (*M* = 200 ms, *SE* = 7) angular degrees, *F*(1, 6) = 1.171, *MSE* = 31.638, *p* = 0.321, η_p_^2^ = .163).

This pattern largely follows that expected from previous reports of the remote distractor effect with saccades slowing as the remote distractor approaches fixation, which supports the suggestion that remote distractor impacts directly on activity at fixation (i.e., it acts by increasing in fixation activity, thereby slowing saccade responses). The analysis also shows that the remote distractor effect increased the further its distance from the target. This supports other results showing evidence that the remote distractor also interacts directly with target activation by contributing to it when closer, thereby speeding up saccade responses (McSorley et al., 2012).

#### Effect of remote distractor distance from fixation and target on average global effect

**Figure 3.**
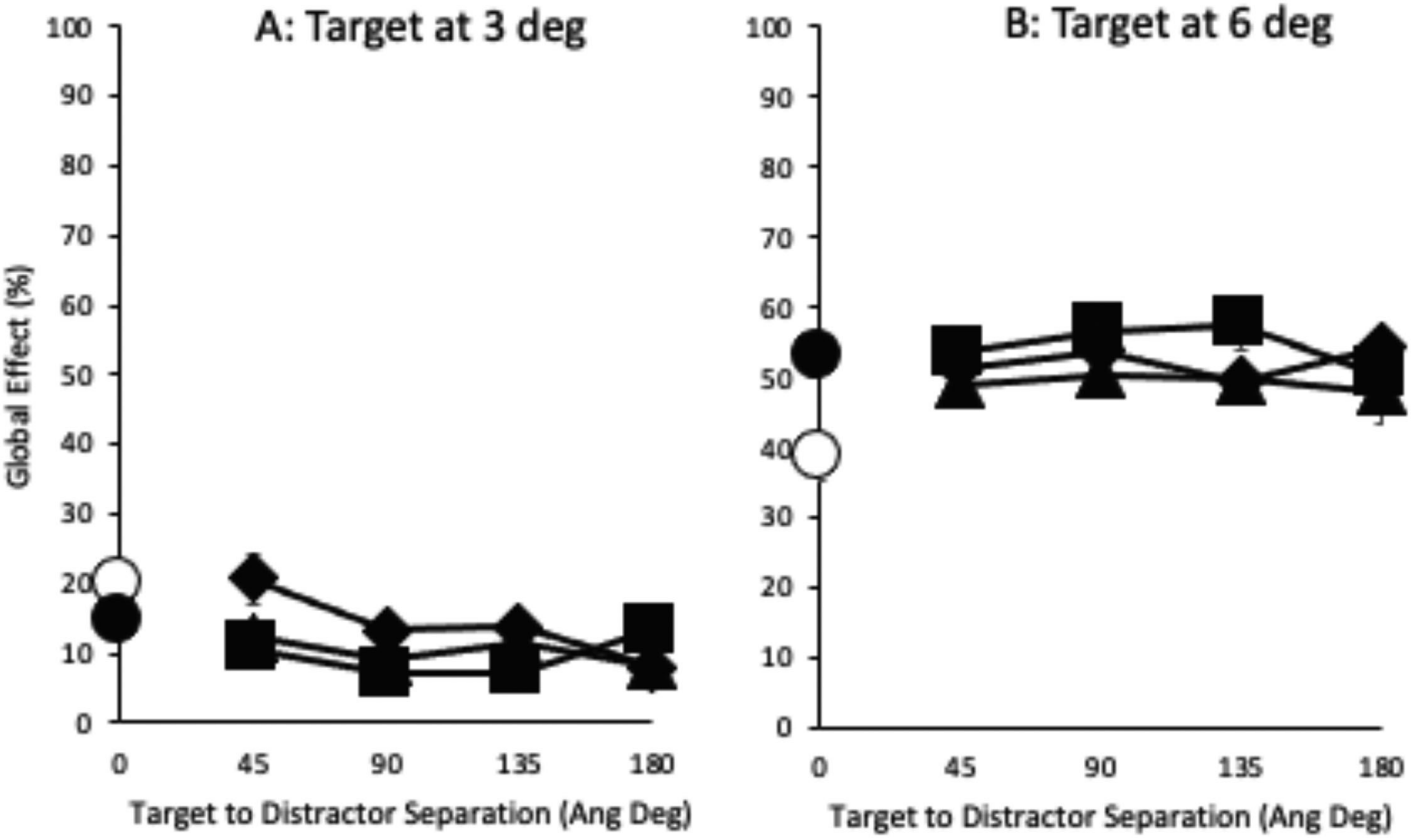
The effect of target and close distractor presence on saccade amplitude as a global effect in which a larger value indicates a greater influence of the close distractor. This is plotted as a function of remote distractor presence and distance from fixation (3, 6, and 9 degrees of visual angle, labeled RD 3, RD 6, and RD 9) and target (45, 90, 135, and 180 angular degrees). The filled black circle on the ordinate shows the global effect for no remote distractor present while the white circle shows the global effect when the remote distractor is shown in the central location. All other positions are plotted as indicated on the abscissa and the legend. Error bars are within-subjects error bars (Cousineau, 2005).

Having established that a remote distractor slows saccade latencies the extent of which depends on distance from fixation and distance from target, we now move on to examine the effect on saccade amplitude. In order to examine this, we derived the global effect for each condition separately in the following way. The average saccade amplitude when each target was presented alone was determined for each participant separately. These were taken as baselines, and the amplitudes for each condition were determined relatively as an index of close distractor influence (see tables in Supplementary Material), with large values indicating greater close distractor influence and lower values a greater influence of the target. At the extreme, then, a 0% global effect showed the same saccade amplitude as evoked when the target was shown alone, and a global effect of 100% showed a similar saccade amplitude as when the close distractor was shown alone (i.e., when it was the single target). In order to compute this, we employed the formula put forward by Findlay, Brogan, and Wenban-Smith (1993), that is, (x-target alone/target alone – close distractor alone) * 100. Figure 3 shows the average global effect across participants for each target and remote distractor position.

As with median saccade latencies, the global effect caused by the presence of the close distractor was examined across remote distractor condition. Initially, by presence: no remote distractor, central remote distractor, and “other” remote distractor position. A two-way ANOVA with target location and remote distractor presence was carried out. This showed main effects of both factors and an interaction (target location, *F*(1, 6) = 46.139, *MSE* = 243.133, *p* < 0.001, η_p_^2^ = .885; remote distractor, *F*(2, 12) = 4.538, *MSE* = 15.132, *p* = 0.034, η_p_^2^ = .431; interaction, *F*(2, 12) = 10.552, *MSE* = 49.45, *p* = 0.002, η_p_^2^ = .638). Simple main effects analysis examining the effect of remote distractor presence for each target location shows that there were no significant differences when the target was at 3 degrees (although center (*M* = 20%, *SE* = 3) vs. other (*M* = 11%, *SE* = 2) was marginal, *p* = 0.052; other *p*s > 0.532). When the target was at 6 degrees, on the other hand, significant differences were found between center (*M* = 39%, *SE* = 6) and none (*M* = 53%, *SE* = 6, *p* = 0.011) and center and other (*M* = 52%, *SE* = 5, *p* = 0.03, other *p*s = 1). This shows that the global effect was not affected by remote distractor presence when the target was close to fixation but reduced when presented centrally when the target was presented at a greater distance.

A three-way ANOVA was carried out next to examine the effect on the global effect of having a remote distractor also present that varied in its distance from fixation (3, 6, and 9 degrees of visual angle) and distance from the target (45, 90, 135, and 180 angular degrees) across target location. This showed a three-way interaction (*F*(6, 36) = 2.389, *MSE* = 44.285, *p* = 0.048, η_p_^2^ = .285). Separate two-way ANOVAs were then carried out examining each target location separately. For the target at 3 degrees with a close distractor at 6 degrees, there were no main effects of either distance from fixation (*F*(2, 12) = 2.67, *MSE* = 58.125, *p* = 0.110, η_p_^2^ = .308) or distance from target (*F*(3, 18) = 2.974, *MSE* = 36.489, *p* = 0.059, η_p_^2^ = .331), but there was a significant interaction (*F*(6, 36) = 2.555, *MSE* = 33.509, *p* = 0.036, η_p_^2^ = .299). Direct contrasts were carried out to examine the cause of the interaction with distance from fixation considered across each distance from the target and vice versa, but no differences were of sufficient strength to show a significant difference. In the second two-way ANOVA, when the target was shown at 6 degrees along with a close distractor at 3 degrees showed no main effects or interaction (distance from fixation: *F*(2, 12) = 2.215, *MSE* = 87.222, *p* = 0.152, η_p_^2^ = .27; distance from target and the interaction: *F*s < 1).

Overall, we can see evidence for a clear global effect in the data, including a clear difference between global effect extent for closer distractors compared with distractors further away. This is similar to the results reported by Coren and Hoenig (1972) and McSorley and Findlay's (2003) near distractor effect. There is some evidence when the target is further away from fixation than a close distractor that a central remote distractor reduces global effect extent, but there is no strong picture showing clear influences of remote distractor position on global effect.

#### Relationship between global effect and remote distractor

**Figure 4.**
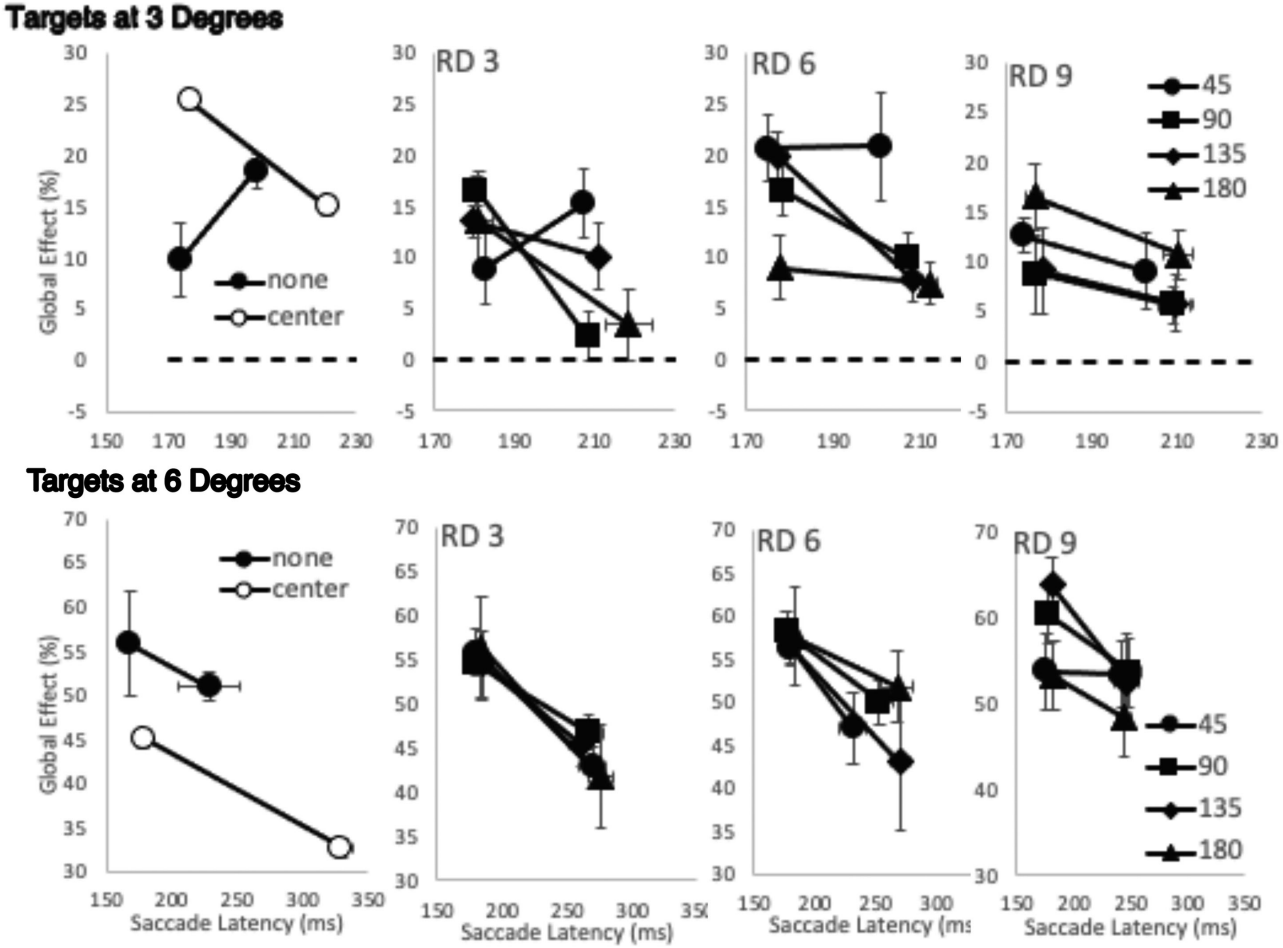
The average global effect as a function of saccade latency after the median split was performed on the basis of the distribution of saccade latencies. The median split was carried out separately for each condition and participant. The global effect found with targets at 3 degrees are shown in the top row and targets at 6 degrees in the bottom row. Note the scale difference for the saccade latencies elicited by each target location. The left column shows data when no remote distractor is present and when it was shown in the central position. Moving rightward across the columns shows the data found when the remote distractor was shown progressively greater distances from fixation (3, 6, and 9 degrees of visual angle, labeled RD 3, RD 6, and RD 9). On each of these graphs, data are shown for each remote distractor distance from the target (45, 90, 135, and 180 angular degrees). Error bars are within-subjects error bars (Cousineau, 2005).

On the face of it, there are very different pattern of effects on saccade latencies and accuracy of the presence and position of the remote distractor. Saccade latencies were found to lengthen as remote distractor approached fixation and increased with distance from the target while saccade accuracy, as indexed through the global effect, showed effects of remote distractor presence, but this is neither strong nor consistently related to remote distractor position. This suggests while the presence and position of the remote distractor clearly impact on saccade initiation mechanisms, they do not play a direct role in those involved in determining saccade amplitude. However, while suggestive, this different overall pattern showing the impact of remote distractor presence on saccade latency and its accuracy does not directly compare the relationship between remote distractor position and global effect. In order to do this, the shorter and longer latency saccades for each remote distractor position were extracted by conducting a median split of the latency distributions for each condition. The average global effect was then determined for the shorter and longer latency saccades. This was carried out separately for each participant. The overall average across participants is shown in Figure 4^5^.

**Figure 5.**
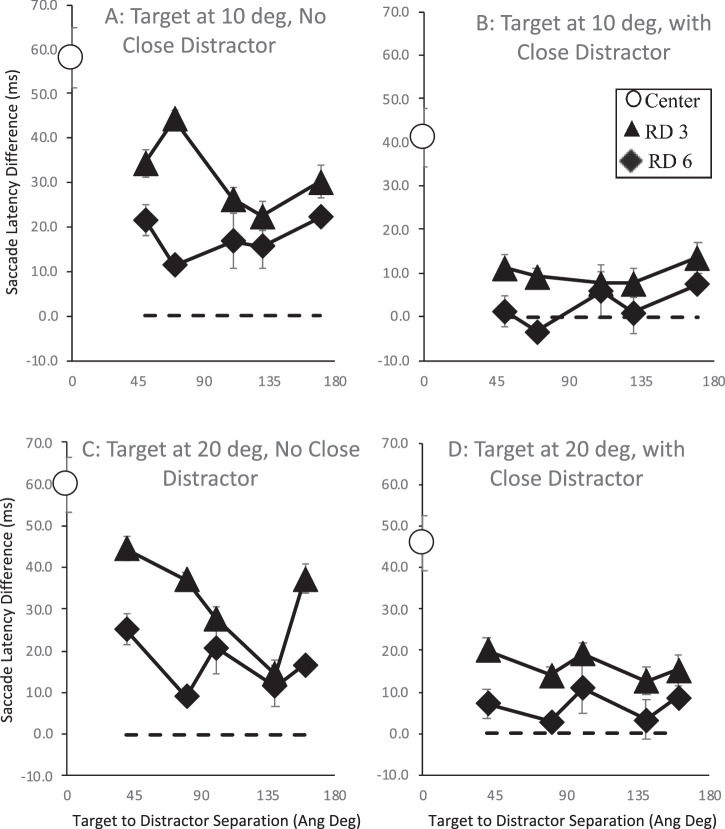
The remote distractor effect as a function of target distance, close distractor presence, and the remote distractor distance from fixation and target. Results when the target and close distractor were presented 6 degrees of visual angle from fixation and 10 angular degrees above or below the horizontal meridian are shown in the upper row. Results when target and close distractor separation was increased with each presented 20 angular degrees above or below the horizontal meridian are shown in the lower row. The effect of the remote distractor is shown for distances from fixation (3 and 6 degrees of visual angle, labeled RD 3 and RD 6) and for each remote distractor distance from the target. Error bars are within-subject error bars (Cousineau, 2005).

A three-way ANOVA was carried out with target location, saccade latency, and remote distractor presence. This showed a main effect of target location (*F*(1, 6) = 46.805, *MSE* = 489.011, *p* < 0.001, η_p_^2^ = .886), a main effect of remote distractor (*F*(2, 12) = 4.087, *MSE* = 29.981, *p* = 0.044, η_p_^2^ = .405), and two-way interactions between target location and remote distractor presence and latency and remote distractor presence (*F*(1, 6) = 10.196, *MSE* = 105.502, *p* = 0.003, η_p_^2^ = .63; *F*(1, 6) = 8.232, *MSE* = 38.797, *p* = 0.006, η_p_^2^ = .578, all other *p*s > 0.177). Simple main effects analysis showed that the interaction between target location and remote distractor presence was due to remote distractor presence, provoking differences in global effect extent for each target location. There were found to be no significant differences in the global effect extent for targets at 3 degrees (*p*s > 0.053), whereas there was a significant reduction in global effect for targets at 6 degrees when the remote distractor was shown centrally compared with no remote distractor and when it was shown at the other position (none (*M* = 53%, *SE* = 6) vs. center (*M* = 40%, *SE* = 7), *p* = 0.012; center vs. other (*M* = 52%, *SE* = 5), *p* = 0.032). The second interaction between saccade latency and remote distractor presence was found to be due to their being no significant difference in global effect between short and long latencies when no remote distractor was present (*p* = 0.656) but both center and other showing significantly reduced global effects for long latency saccades compared with shorter latency responses (other: longer latencies, *M* = 29%, *SE* = 4 vs. shorter latencies, *M* = 35%, *SE* = 3, *p* = 0.002; center: longer latencies, *M* = 24%, *SE* = 3 vs. shorter latencies, *M* = 35%, *SE* = 4, *p* = 0.028). Further contrasts for short latency responses alone show no difference in global effect extent regardless of remote distractor presence (*p*s > 0.997). Longer latency responses show a significantly smaller global effect for centrally presented remote distractor conditions (none (*M* = 35%, *SE* = 5) vs. center (*M* = 24%, *SE* = 3), *p* = 0.015; center (*M* = 24%, *SE* = 3) vs. other (*M* = 29%, *SE* = 4), *p* = 0.023), with global effect extent being largest when no remote distractor was present and becoming less as the remote distractor was present in the other location and then in the central location.

These contrasts mirror those reported in the main global effect analysis in the preceding section, in that the global effect elicited when targets are close to fixation is little affected by the presence of a remote distractor, while the central remote distractor reduces global effect extent when the target is further away. But here we also show that targeting closer targets is little affected by the latency of the response, while the global effect evoked toward targets further away when a central remote distractor is present is dependent on saccade latencies.

To explore the effect of remote distractor distance from fixation and from target (an unpacking of the “other” position), a four-way ANOVA was carried out with target location, latency, remote distractor distance from fixation, and remote distractor distance from target as factors. This showed significant main effects for all factors except for distance from the target (target location, *F*(1, 6) = 107.636, *MSE* = 1,312.688, *p* < 0.001, η_p_^2^ = .947; latency, *F*(1, 6) = 8.229, *MSE* = 465.348, *p* = 0.028, η_p_^2^ = .578; distance from fixation, *F*(2, 12) = 4.328, *MSE* = 70.162, *p* = 0.038, η_p_^2^ = .419). There was a three-way interaction between target location, distance from fixation, and distance from target (*F*(6, 36) = 2.526, *MSE* = 87.358, *p* = 0.038, η_p_^2^ = .296) and a significant four-way interaction (*F*(6, 36) = 2.452, *MSE* = 38.341, *p* = 0.043, η_p_^2^ = .29).

To examine this further, separate three-way ANOVAs were carried out for each target location. For the 3-degree target, there were no main effects, but there was a significant two-way interaction of remote distractor distance from fixation and remote distractor distance from target (*F*(6, 36) = 2.554, *MSE* = 68.813, *p* = 0.037, η_p_^2^ = .299, and a three way interaction, *F*(6, 36) = 2.368, *MSE* = 48.909, *p* = 0.050, η_p_^2^ = .283). However, a simple main effects analysis to explore this further showed no comparisons that statistically survived further exploration, showing a lack of general pattern to the effect of remote distractor. The second three-way ANOVA examining the global effect when the target was shown at 6 degrees showed only a main effect of latency (*F*(1, 6) = 13.469, *MSE* = 237.74, *p* = 0.01, η_p_^2^ = .693), displaying a reduction in global effect for longer saccades regardless of remote distractor position (longer latency global effect, *M* = 57%, *SE* = 5; shorter latency global effect, *M* = 48%, *SE* = 6).

Overall, the results showed an effect of the remote distractor on saccade latencies, with a general slowing in its presence that depended on its proximity to fixation and the target. There was also a global effect for both targets, but only the global effect evoked by the 6-degree target and 3-degree close distractor was found to be affected by remote distractor presence and by saccade latency. Closer inspection of the relationship between remote distractor, latency, and global effect showed little evidence that the position of the remote distractor played a role in this; rather, an effect of latency seemed to dominate. Thus, although there are reasons to be cautious, on the whole, the results do support the position that the impact of a remote distractor, when found, is more simply explained by the lengthening of saccade latency, which allows an improvement in saccade accuracy.

## Experiment 2

### Off-axis target locations of 10 and 20 degrees

#### Median saccade latencies

In Experiment 2, the targets were presented “off-axis,” 6 degrees of visual angle from fixation at either 10 and 20 angular degrees. The analysis strategy followed that pursued in Experiment 1.

#### Effect of remote distractor presence on median saccade latencies

As with Experiment 1, separate two-way ANOVAs were carried out depending on close distractor presence with target location (10 degrees and 20 degrees) and remote distractor presence (no remote distractor, remote distractor at center, and a further grouped condition of remote distractor in an “other” position) as factors. With no close distractor present, only a main effect of remote distractor presence was found (*F*(2, 12) = 29.929, *MSE* = 409.940, *p* < 0.001, η_p_^2^ = .833, other *p*s > 0.108), whereas when a close distractor was present, main effects of target location and remote distractor presence were found (target location, *F*(1, 6) = 11.906, *MSE* = 137.27, *p* < 0.014, η_p_^2^ = .665; remote distractor presence, *F*(2, 12) = 26.065, *MSE* = 283.524, *p* < 0.001, η_p_^2^ = .813, other *p*s > 0.632). The main effect of target location showed that saccades evoked by targets at 20 degrees had longer latencies. Follow-up contrasts exploring the effect of remote distractor presence were conducted examining center versus no remote distractor, other versus no remote distractor, and center versus other. These show that the presence of both the central and other remote distractor induced a slowing of the saccade indicative of a remote distractor effect, which was largest when it was shown at the center (no close distractor: center (*M* = 210 ms, *SE* = 11) vs. none (*M* = 151 ms, *SE* = 4), *F*(1, 6) = 33.948, *MSE* = 1,432.071, *p* = 0.001, η_p_^2^ = .850; other (*M* = 176 ms, *SE* = 4) vs. none, *F*(1, 6) = 51.820, *MSE* = 162.167, *p* < 0.001, η_p_^2^ = .896; center vs. other, *F*(1, 6) = 19.176, *MSE* = 865.405, *p* = 0.005, η_p_^2^ = .762; close distractor: center (*M* = 205 ms, *SE* = 8) vs. none (*M* = 161 ms, *SE* = 2), *F*(1, 6) = 30.167, *MSE* = 878.167, *p* = 0.002, η_p_^2^ = .834; other (*M* = 170 ms, *SE* = 3) vs. none, *F*(1, 6) = 25.324, *MSE* = 44.071, *p* = 0.002, η_p_^2^ = .808; center vs. other, *F*(1, 6) = 21.482, *MSE* = 778.905, *p* = 0.004, η_p_^2^ = .782).

#### Effect of remote distractor distance from fixation and target on median saccade latencies

As with Experiment 1, three-way ANOVAs were performed to examine the effect of remote distractor distance from fixation and distance from target with target location as an additional factor (see Figure 5). Separate ANOVAs were carried out on the basis of close distractor presence. In either case, there were only main effects (no interactions) found for target location and remote distractor distance from fixation, with longer latencies when target and close distractor were more widely separated and with remote distractors shown closer to fixation, respectively (with no close distractor present: target location (10-degree target, *M* = 180 ms, *SE* = 5; 20-degree target, *M* = 171 ms, *SE* = 4), *F*(1, 6) = 22.099, *MSE* = 130.74, *p* = 0.003, η_p_^2^ = .786; distance from fixation (3-degree remote distractor, *M* = 183 ms, *SE* = 6; 6-degree remote distractor, *M* = 169 ms, *SE* = 4), *F*(1, 6) = 9.914, *MSE* = 767.331, *p* = 0.02, η_p_^2^ = .623; all other *p*s > 0.052; with close distractor present: target location (10-degree target, *M* = 163 ms, *SE* = 3; 20-degree target, *M* = 177 ms, *SE* = 4), *F*(1, 6) = 27.566, *MSE* = 266.424, *p* = 0.002, η_p_^2^ = .821; distance from fixation (3-degree remote distractor, *M* = 174 ms, *SE* = 4; 6-degree remote distractor, *M* = 166 ms, *SE* = 3), *F*(1, 6) = 24.545, *MSE* = 104.762, *p* = 0.003, η_p_^2^ = .804; all other *p*s > 0.121).

#### Effect of remote distractor distance from fixation and target on average global effect

**Figure 6.**
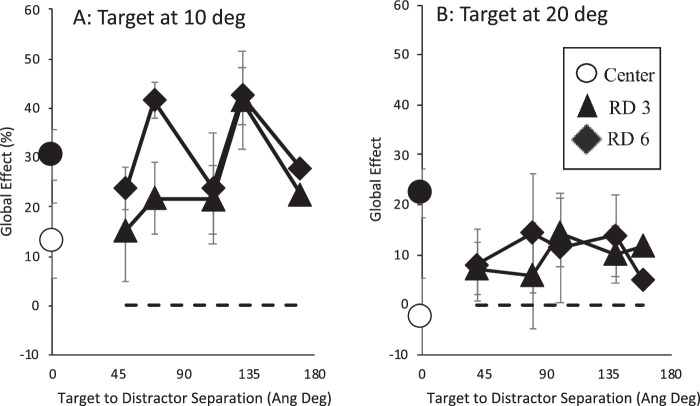
The effect of target and close distractor presence on saccade direction as a global effect in which a larger value indicates a greater influence of the close distractor. This is plotted as a function of remote distractor presence and distance from fixation and target. The effect of the remote distractor is shown for distances from fixation (3 and 6 degrees of visual angle, labeled RD 3 and RD 6) and for each remote distractor distance from the target. The filled black circle on the ordinate shows the global effect for no remote distractor present while the white circle shows the global effect when the remote distractor is shown in the central location. All other positions are plotted as indicated on the abscissa and the legend. Error bars are within-subjects error bars (Cousineau, 2005).

As with Experiment 1, in order to examine the effect of remote distractor presence on saccade accuracy, we derived the global effect for each condition separately. First, the average landing position when each target was presented alone was determined by extracting saccade direction (as opposed to saccade amplitude in Experiment 1) for each participant separately. These were taken as baselines, and saccade direction for each condition was determined as an index of close distractor influence, with large values indicating greater close distractor influence and lower values a greater influence of the target. As in Experiment 1, global effect percentage was derived in the same manner suggested by Findlay et al. (1993). At the extreme, then, a 0% global effect showed the same saccade direction as when the target was shown alone and a global effect of 100% as having the same as when the close distractor was shown alone (i.e., when it was the single target). The overall global effect of target and close distractor presence averaged across participant is shown in Figure 6.

The global effect caused by the presence of the close distractor was examined across target location and remote distractor condition. A two-way ANOVA showed a significant effect of remote distractor presence only (*F*(2, 12) = 15.17, *MSE* = 105.693, *p* = 0.001, η_p_^2^ = .717). Direct contrasts revealed that the central remote distractor decreased the global effect (none (*M* = 26%, *SE* = 6) vs. center (*M* = 5%, *SE* = 5), *F*(1, 6) = 30.441, *MSE* = 101.958, *p* = 0.001, η_p_^2^ = .835; center vs. other (*M* = 19%, *SE* = 3), *F*(1, 6) = 9.105, *MSE* = 147.509, *p* = 0.023, η_p_^2^ = .603). In order to examine the effect of remote distractor distance from fixation and from target, the factor “other” was unpacked and a three-way ANOVA was carried out. This revealed only a main effect of target location, with targets 10 degrees from the horizontal evoking a greater global effect than the more widely separated target–close distractor pair (10-degree target (*M* = 28%, 5) vs. 20-degree target (*M* = 10%, *SE* = 2), *F*(1, 6) = 16.46, *MSE* = 691.365, *p* = 0.007, η_p_^2^ = .733, all other *p*s > 0.217).

As with Experiment 1, with on-axis targets, there is little evidence that global effect is affected by the position of the remote distractor (across-differences distances from fixation and the target), but there is a clear reduction in global effect when a remote distractor is presented in a central position.

#### Relationship between global effect and remote distractor

**Figure 7.**
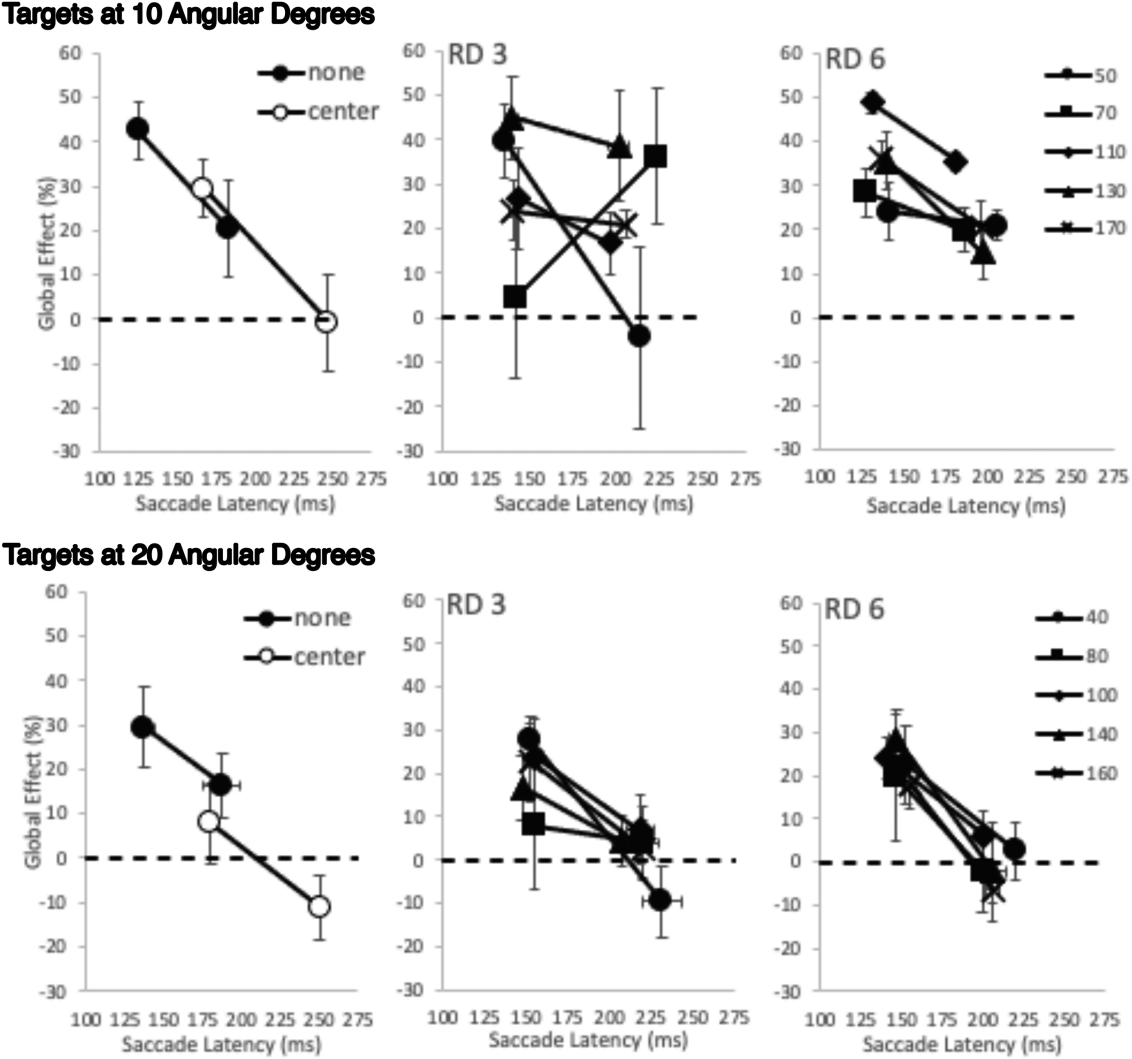
The average global effect as a function of saccade latency after the median split was performed on the basis of the distribution of saccade latencies. The median split was carried out separately for each condition and participant. The global effect found with targets at 10 degrees are shown in the top row and targets at 20 degrees in the bottom row. The left column shows data when no remote distractor is present and when it was shown in the central position. Moving rightward across the columns shows the data found when the remote distractor was shown progressively greater distances from fixation (3 and 6 degrees of visual angle, labeled RD 3 and RD 6). On each of these graphs, data are shown for each remote distractor distance from the target (shown on the right-hand side of each row in the legend; these distances are in angular degrees). Error bars are within-subjects error bars (Cousineau, 2005).

Next we directly compare the relationship between remote distractor position and global effect. The shorter and longer latency saccades for each remote distractor position were extracted by conducting a median split of the latency distributions for each condition. The average global effect was then determined for the shorter and longer latency saccades. This was carried out separately for each participant. The cross-participant average is shown in Figure 7.

A three-way ANOVA was carried out to examine the global effect as a function of saccade latency (short and long) remote distractor presence (none, center, other) across target location. This showed a main effect of latency and remote distractor presence only (latency, *F*(1, 6) = 14.119, *MSE* = 543.757, *p* = 0.009, η_p_^2^ = .702; remote distractor presence, *F*(2, 12) = 13.785, *MSE* = 226.446, *p* = 0.001, η_p_^2^ = .697, other *p*s > 0.185). The main effect of saccade latency is due to shorter latencies evoking a larger global effect (shorter latency global effect *M* = 27%, *SE* = 3; longer latency global effect, *M* = 8%, *SE* = 6). Further contrasts examining remote distractor presence show a significant reduction in global effect when the remote distractor was shown in the central position (center (*M* = 6%, *SE* = 5) vs. none (*M* = 27%, *SE* = 5), *F*(1, 6) = 26.301, *MSE* = 461.172, *p* = 0.002, η_p_^2^ = 814; center vs. other (*M* = 20%, *SE* = 3), *F*(1, 6) = 8.091, *MSE* = 630.366, *p* = 0.029, η_p_^2^ = .574).

Unpacking analysis of remote distractor positions across target location, saccade latency, remote distractor from distance from fixation, and remote distractor from target in a four-way ANOVA shows a main effect of target location and latency and an interaction between them (target location: *F*(1, 6) = 14.791, *MSE* = 466.786, *p* = 0.008, η_p_^2^ = .711; saccade latency: *F*(1, 6) = 21.893, *MSE* = 745.702, *p* = 0.003, η_p_^2^ = .785; target location by saccade latency, *F*(1, 6) = 8.859, *MSE* = 203.021, *p* = 0.025, η_p_^2^ = .596, all other *p*s > 0.093). These reflect the overall pattern of global effect dependencies on target location and latency, with a larger global effect being shown when the target and close distractor are closer together and saccade latencies are short (shorter latency to 10-degree target global effect, *M* = 34%, *SE* = 5; longer latency to 10-degree target global effect, *M* = 24%, *SE* = 5; shorter latency to 20-degree target global effect, *M* = 21%, *SE* = 3; longer latency to 20-degree target global effect, *M* = 1%, *SE* = 3). Exploration of the interaction showed that the effect of latency on the global effect was found regardless of target–close distractor separation (10 degrees (shorter latency to 10-degree target global effect, *M* = 34%, *SE* = 5; longer latency to 10-degree target global effect, *M* = 24%, *SE* = 5), *F*(1, 6) = 11.755, *p* = 0.014, η_p_^2^ = .622; 20 degrees (shorter latency to 20-degree target global effect, *M* = 21%, *SE* = 3; longer latency to 20-degree target global effect, *M* = 1%, *SE* = 3), *F*(1, 6) = 22.669, *p* = 0.003, η_p_^2^ = .791). The cause of the interaction was a significant reduction in global effect for longer latency responses between targets at 10 degrees and 20 degrees but not the shorter latencies (longer latency to 10-degree target global effect (*M* = 24%, *SE* = 5) vs. longer latency to 20-degree target global effect (*M* = 1%, *SE* = 3), *F*(1, 6) = 30.960, *p* = 0.001, η_p_^2^ = .838). Taken together, these suggest that saccade targeting improves with a longer response time (as would be expected), and this improvement is greater when the target is more widely separated from the close distractor.

Overall, then, and in line with the findings of Experiment 1, the results show an effect of the remote distractor presence on saccade latency (the remote distractor effect) and an effect of the presence of the close distractor on saccade direction (the global effect). Beyond these basic effects, there is some evidence that remote distractor presence influences saccade accuracy, with reductions in global effect being found in the presence of the remote distractor in the center. But the main finding from consideration of the median split latency analysis is that increases in saccade latency reduce the global effect. This, along with Experiment 1 and previous reports (Cruickshank & McSorley, 2009; McSorley & Cruickshank, 2010), supports the suggestion that the impact of remote distractor on the control of saccade direction is mostly via indirect mechanisms that interfere with fixation disengagement (possibly through an increase in activity at fixation), which allows saccade targeting mechanisms to continue unabated and become more firmly centered on the target.

## The effect of the ratio of distractor to target eccentricity in Experiment 1 and Experiment 2

Analysis examining the interaction between the close and remote distractor effects on saccade latency and amplitude has been concentrated on exploring the effects of remote distractor distance from fixation and distance from target separately. However, a further common approach to examining the remote distractor effect has been to consider them in terms of remote distractor distance from fixation as a ratio of target distance from fixation. In this way, the effect of the stimuli distance from fixation can be isolated. For example, when the remote distractor and target are presented 6 degrees from fixation, then they have a ratio of 1 regardless of angular distance of the remote distractor from the target. As noted in the Introduction, the remote distractor effect has been shown to be a function of this ratio, progressively increasing as the ratio decreases (i.e., increasing as the distance of the remote distractor approaches fixation). It may be the case that the differences found in the effect of remote distractor distance from fixation and from the target on saccade latency and amplitude reported here can be better captured when considered in terms of this ratio.

**Figure 8.**
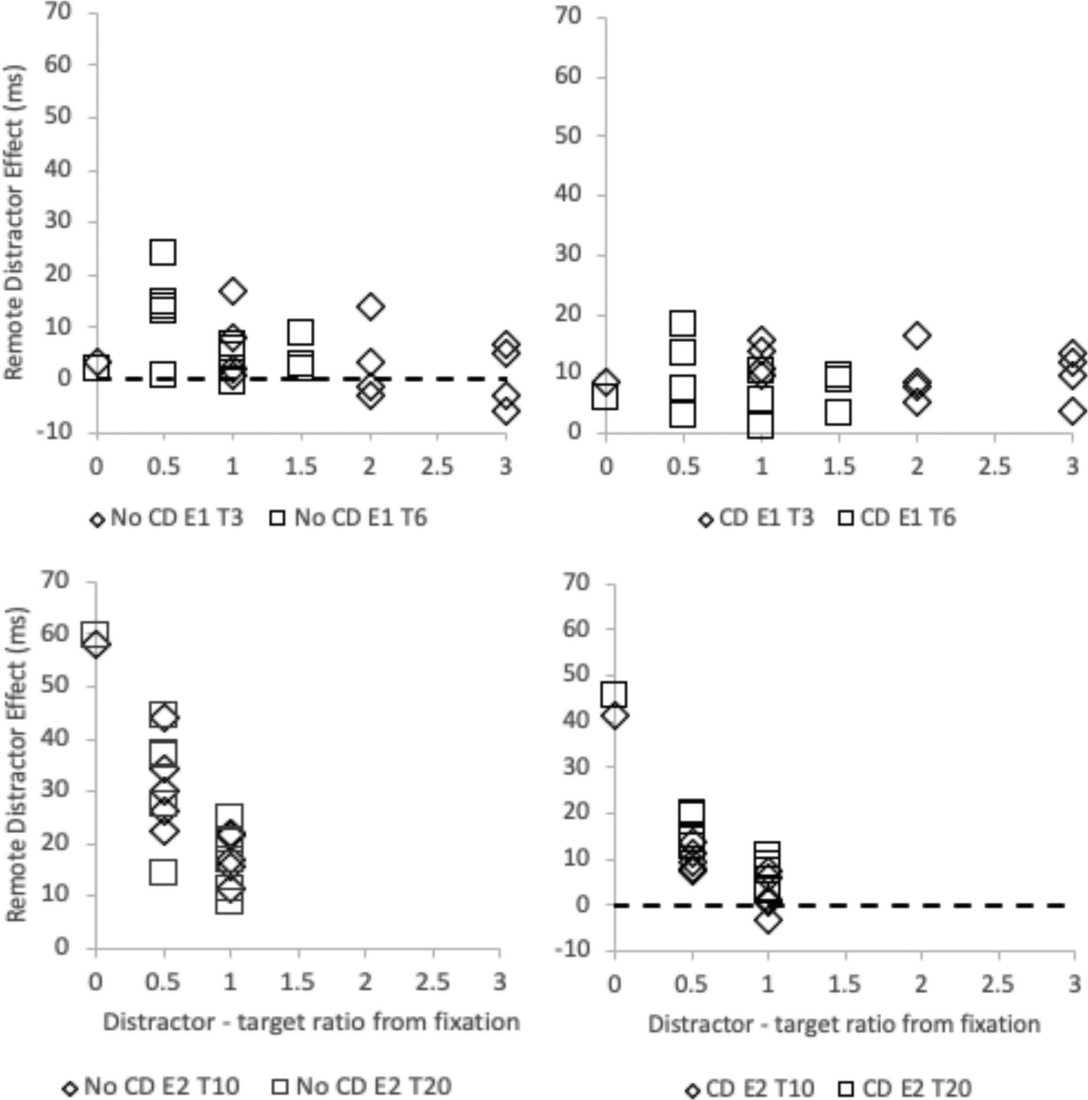
The remote distractor effect in milliseconds (ms) as a function of the ratio of the remote distractor distance from fixation relative to the remote distractor distance from target. Results from Experiment 1 (E1) are shown in the upper row and results from Experiment 2 (E2) are shown in the lower row. The left-hand column shows conditions when a close distractor was not present while the right-hand column shows results when the close distractor was present.

**Figure 9.**
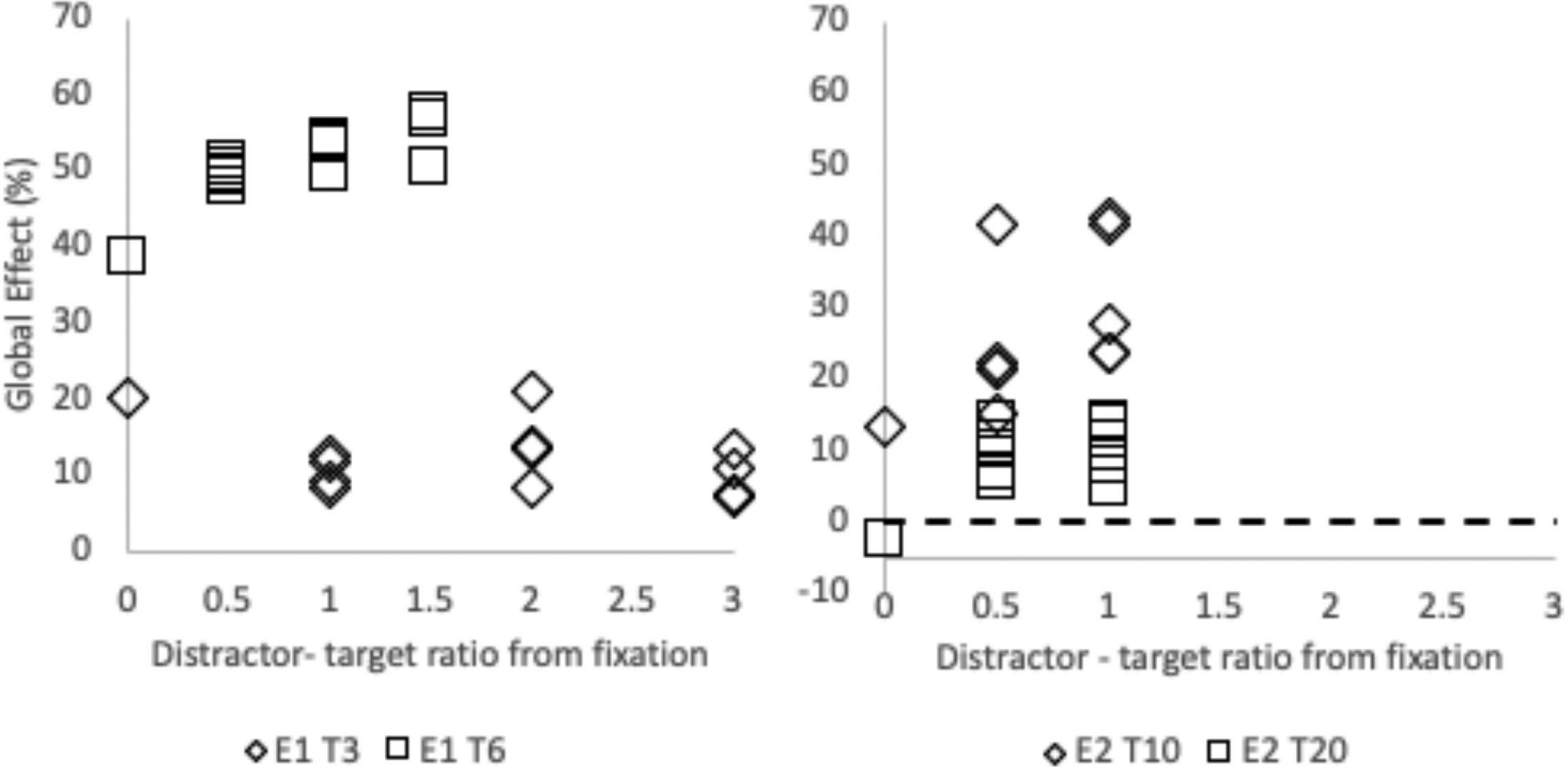
The global effect (as a percentage) as a function of the ratio of the remote distractor distance from fixation relative to the remote distractor distance from target. Results from Experiment 1 (E1) are shown in left panel and those from Experiment 2 (E2) are presented in the right panel.

In order to examine this, the remote distractor effect and global effect from Experiment 1 and Experiment 2 were replotted in terms of the ratio of distractor to target eccentricity and are shown in Figure 8 and Figure 9. The impact of this ratio on the remote distractor effect when the close distractor is or is not present was examined separately. Due to clear main effect differences between the global effect induced by the close distractor for each target location (as can be seen in Figure 9), ratio effects were examined for each of these separately.

This analysis shows an effect of ratio on remote distractor effect, which mirrors that reported by Walker et al. (1997) and Casteau and Vitu-Thibault (2012): As the ratio decreases and the remote distractor is situated more closely to fixation than the target, the remote distractor effect extent increases. Indeed, correlation analysis shows a relationship between ratio extent and remote distractor effect extent in Experiment 1 when the close distractor is not present and in Experiment 2 regardless of the presence of the close distractor (Experiment 1, no close distractor: *r*(26) = –.402, *p* = 0.042; Experiment 1, with close distractor: *r*(26) = .078, *p* = 0.706; Experiment 2, no close distractor: *r*(22) = –.846, *p* < 0.001; Experiment 2, with close distractor: *r*(22) = –.846, *p* < 0.001; note that both *r* values for Experiment 2 are the same). These results provide partial support (in three of the four ratios calculated here) for the suggestion that the extent of the remote distractor effect is a function of the relative eccentricity of the stimuli.

The same analysis was then carried out with global effect extent and showed little evidence supporting a relationship of global effect extent with the remote distractor to fixation and target to fixation ratio. Correlation analysis shows a relationship between ratio extent and global effect extent only in Experiment 1, when the target is shown at 6 degrees from fixation (Experiment 1, 6 degrees: *r*(13) = –.792, *p* = 0.001). There were no other correlations for the other target positions (all *p*s > 0.078). Contrary to the relationship found for remote distractor effect with the stimuli eccentricity ratio, these results provide little support that the extent of the global effect is a function of the relative eccentricity of the stimuli.

Overall, this pattern provides support for the position that the remote distractor primarily impacts activation at fixation and acts to interrupt disengagement. It increases saccade latency but does not directly impact on saccade amplitude. The results examining saccade latency and global effect suggest that saccade amplitude is more directly tied to interactions between the target and nearby distractors and saccade latency rather than any direct effect of the remote distractor.

## Discussion

The examination of the relationship between saccade latency and global effect shows little to support the suggestion that the remote distractor impacted directly on the interactions between the target and closer distractor. Rather, the results support a more indirect interaction with the remote distractor impairing fixation disengagement, allowing saccade targeting processes to continue unabated and be accessed later and hence result in more accurate saccades. Both experiments presented here show accuracy improvements (in amplitude, Experiment 1; direction, Experiment 2) associated with the presence of a remote distractor that largely follow a similar pattern regardless of the presence of a close distractor and regardless of the position of the remote distractor. This conclusion is based on our findings showing a global effect and a remote distractor effect with little evidence showing a dependence between them. Saccade accuracy was affected by the presence of a distractor presented close to the target. Its magnitude was found to be dependent on saccade latency. We will now consider these three critical findings in more detail, that is, (a) the global effect, (b) the remote distractor effect, and (c) the lack of dependency between them.

*(a) The* *global effect: Saccades are less accurate when made in the presence of a distractor close to a target.*

As would be expected, the presence of a distractor close to the target location was found to influence saccade accuracy as estimated based on saccade amplitude in Experiment 1 and saccade direction in Experiment 2, with the increase in the global effect showing that saccades land between the target and close distractor position compared with a similarly located target shown in isolation (e.g., Deubel et al., 1984; Findlay, 1982; He & Kowler, 1989; McSorley et al., 2012; van der Stigchel et al., 2007; Walker et al., 1997). This suggests that within a certain spatial window, representations of, and hence underlying neural activation caused by, visual stimuli are pooled and contribute to determining the computation of saccade metrics (Glimcher & Sparks, 1993; Lee et al., 1998; McIlwain, 1986, 1991; Ottes et al., 1984, 1985; van Opstal & van Gisbergen, 1989).

The global effect is usually attributed to distributed spatial coding in the neural populations in the intermediate and deeper layers of SC, which respond to large and overlapping receptive or movement fields (Edelman & Keller, 1998; van Opstal & van Gisbergen, 1989). These respond retinotopically to visual space with contiguous areas represented across multiple receptive fields centered on adjoining locations (Phongphanphanee et al., 2014; Vokoun, Huang, Jackson, & Basso, 2014). Salient target locations and distractors that are in close proximity elicit activation across many of the neurons in the deep and intermediate layers of SC, and hence saccades are directed to those that represent visual locations somewhere between the targets (Findlay & Walker, 1999; van Opstal & van Gisbergen, 1989). This has been suggested to be a function of an interplay between short-range and long-range connections across SC, which operate in a Mexican hat–style arrangement (Hafed, 2018). Alternatively, it has also been suggested that the global effect arises from the weighted average of the entire active population in SC (Lee, Rohrer, & Sparks, 1998; Meeter et al., 2010; Robinson, 1972).

Our results show that longer latency saccades show a general reduction in the global effect of the close distractor on saccade accuracy. The relationship between remote distractor, latency, and global effect shows little evidence that the position of the remote distractor plays a role in this; rather, the effect of latency seems to dominate. Of course, not all data support this conclusion. There are a large number of conditions here, and it is to be expected that a completely supportive set of data would throw up some unexpected findings. However, on the whole, data are either supportive of, or at least do not commonly run against, the conclusion that the global effect is dependent both on saccade latency and the relationship between closely located stimuli. They are not dependent on the presence of remotely located stimuli. This, along with previous reports (Cruickshank & McSorley, 2009; McSorley & Cruickshank, 2010), supports the suggestion that the impact of remote distractor on the control of saccade amplitude/direction is mostly via indirect mechanisms that interfere with fixation disengagement (possibly through an increase in activity at fixation), which allows saccade-targeting mechanisms to continue unabated and become more firmly centered on the target.

*(b) The* *remote distractor:* *Saccade latency is slowed when made in the presence of a remote distractor,* *and the extent of this depends on its position.*

Results show an increase in saccade latencies in the presence of the remote distractor, the extent of which depended on its position (Godijn & Theeuwes, 2002; McSorley, Cruickshank, & Inman, 2009; McSorley, Haggard, & Walker, 2004, 2009; Van der Stigchel, Meeter, & Theeuwes, 2007; Walker et al., 1997). In general, across both experiments and most conditions, the remote distractor effect was found to increase as the remote distractor was shown closer to fixation. Notably, the remote distractor effect for on-axis targets in Experiment 1 was found to depend on distance from fixation and distance from target (Khan, Munoz, Takahashi, Blohm, & McPeek, 2016; McSorley et al., 2012), but generally, the common finding was that the remote distractor effect was largely a function of distance from fixation.

In terms of the models put forward in the Introduction, the results largely favor an indirect concept of saccade latency control, one in which the presence of a remote distractor impacts directly on saccade latency and only indirectly affects saccade accuracy. Our results support the suggestion that remote distractor operates through competitive interactions between a fixate system and a move system, which hampers fixation disengagement, thus slowing saccade initiation by increasing activation at fixation (Casteau & Vitu-Thibault, 2012; Findlay & Walker, 1999), that is, it impacts on target activation only indirectly through the reciprocal activity between fixate and move systems. The remote distractor increases saccade latency by enhancing activation at fixation-related activity through SC projections to OPN further downstream in the brainstem (Casteau & Vitu-Thibault, 2012; Veale et al., 2017). This increases the length of time needed for target activation to inhibit fixation-related activation. This has been suggested to operate via an indirect extended fixation zone, which acts as a gating system to saccade execution (Gandhi & Keller, 1999). Thus, the remote distractor effect reported here can be ascribed to the function of remote distractor distance from fixation or perhaps, more generally, to the ratio of the relative eccentricity of target and remote distractor from fixation, as suggested by the analysis shown in Figure 8 (see also Casteau & Vitu-Thibault, 2012; Walker et al., 1997).

It also worth bearing in mind that the latency of a saccade also depends on many other target and distractor properties, task demands, and stimuli properties (Coren & Hoenig, 1972; Coëffé & O'Regan, 1987; Kalesnykas & Hallett, 1994; Ludwig, Gilchrist, & McSorley, 2005b). For example, Casteau and Vitu-Thibault (2012) report experiments that involve target discrimination tasks that introduce task demands not commonly encountered in remote distractor effect experiments. Furthermore, saccade latencies will also be dependent on how willing participants are to make saccades. Saccade responses can be slowed or speeded depending on internal motivation or external instruction: After all, we can withhold our saccade responses regardless of how attractive a peripheral target may be. Experimental instructions that emphasize accuracy over speed, or vice versa, can change response times.

*(c) The* *global effect* *does not depend on the* *remote distractor effect: Saccade accuracy is a function of its latency rather than remote distractor presence.*

The relationship between remote distractor effect and global effect was examined by extracting the median split of saccade latency distributions for each remote distractor condition. Despite the overall change in the size of the remote distractor effect depending on remote distractor position, it was found that saccades executed with similar latencies (i.e., similar extents of remote distractor effect) had similar accuracy (i.e., similar extents of global effect). While this was not always the case across all conditions employed here, it was more often found than not and did not run in the opposite direction (i.e., smaller global effect with shorter latency saccades). These results suggest that distractors displayed close to a target impact directly on competing mechanisms involved in saccade target selection. Their presence contributes to the computation of the saccade landing position (and likely saccade trajectory control) via saccade-targeting mechanisms. These have been generally explained in terms of competitive and cooperative mechanisms via inhibitory and excitatory connections in SC detailed in the global effect section (a), whereas remote distractors play a more indirect role in the control of saccade targeting by impacting on fixation disengagement only and not at all on target–close distractor interactions as detailed in the remote distractor effect section (b). Thus, the impact of remote distractors is shown through longer latency saccades, which have more accurate landing positions than shorter latency responses.

In both experiments presented here, we found that accuracy improvements associated with the presence of a remote distractor follow a similar pattern regardless of the presence of a close distracter and regardless of the position of the remote distractor. Previous literature had suggested two possible mechanisms to account for the remote distractor effect: The remote distractor increases saccade latency by invoking activity at fixation, making disengagement more difficult (put forward by Walker et al., 1997); alternatively, the remote distractor interacts with processing of the target and close distracter through a competition between saccade-related neurons (Dorris et al., 2007; Khan et al., 2016; Olivier et al., 1999). The results from the two experiments reported in this article favor the former explanation as the development of saccade target selection does not seem to be strongly influenced by the presence and position of a remote distractor.

Studies of the neural basis of target selection and saccade generation have suggested that the neurons coding potential targets compete as the sensory evidence that supports their location changes or accumulates over time (Glimcher & Sparks, 1993; Gold & Shadlen, 2007; Schall, 2015; Smith & Ratcliff, 2004). Models of this system, although differing in their precise details, all suggest that evidence supporting target selection is integrated over time until some threshold is exceeded (Carpenter, Reddi, & Anderson, 2009; Kopecz, 2003; Meeter et al., 2010; Purcell et al., 2010, Ratcliff et al., 2007; Smith & Ratcliff, 2004; Trappenberg et al., 2001). When there are a number of potential targets present, these compete through mutually inhibitory connections and race toward that threshold. These models have been very successful at accounting for some aspects of the latency of saccadic responses and, more recently, in accounting for saccade landing position and trajectory deviations (Aral & Keller, 2005; Godijn & Theeuwes, 2002; Kopecz, 2003; Marino et al., 2012; Meeter et al., 2010; Satel et al., 2011; Trappenberg et al., 2001; Walton, Sparks, & Gandhi, 2005; Wilimzig, Schneider, & Schöner, 2006). However, while some of these models (e.g., Meeter et al., 2010; Trappenberg et al., 2001) predict that a close distractor impacts on landing position while speeding latency (Walker et al., 1997) and that as the distractor is presented further from the target, saccade latency increases, they do not account for the changes found in saccade latency or landing position found here (or those reported by McSorley et al., 2012; Walker et al., 1997) when the remote distractor position is varied. In fact, it is difficult to see how the saccade accuracy improvements we find in the presence of the remote distractor could be due to interactions in saccade target selection processes. There are no changes in accuracy across each remote distractor condition, suggesting that the development of target selection progresses at the same pace regardless of whether the remote distractor is present and regardless of where it is located. Instead, the results favor the explanation that saccade accuracy is improved by the presence of the remote distractor (Cruickshank & McSorley, 2009; McSorley & Cruickshank, 2010; McSorley & Findlay, 2003) as part of a general continuum on which increased saccade latency ordinarily gives improved accuracy whether the remote distractor is present or not. This suggests that the effect of a remote distractor is to increase saccade latency only, with no extra influence on saccade target selection processes.

One potential problem with this explanation is that the latency effect caused by the remote distractor changes has been shown to be both dependent on distance from fixation and, on occasion, distance from target (here shown in Experiment 1 but also reported by McSorley et al., 2009, 2012; however, cf. Walker et al., 1997). If, as we suggest, the latency increase caused by the presence of the remote distractor is solely due to its impact on activity at fixation, why does latency change as the distance from the target increases? Such a pattern points to the remote distractor having a more direct impact on the development of the target signal used to direct the saccade. However, counter to this, we see no evidence of this in the accuracy profile. Furthermore, this dependency on remote distractor distance is not seen in Experiment 2, in which the target–close distractor stimuli are shown off the horizontal axis. In order to account for this, we can speculate that there may be differences in remote distractor effect between the two experiments that provides an explanation. One fundamental difference between Experiment 1 and Experiment 2 is the changing uncertainty about target locations and area over which a target can potentially appear. While in both experiments, the target stimuli are tied to one dedicated direction (left or right), in Experiment 1, they can appear in only two locations both on the horizontal axis, whereas in Experiment 2, there are four potential target locations spread over a larger window of at least 20° (angular degrees; center to center). It may be this difference that changes the approach adopted by our observers. In performing the task, observers are told where targets may appear and asked to ignore all distracters. We suggest they do this by inhibiting nonpotential target locations throughout the experiment and, further, that this inhibitory process is centered on potential target locations such that it is strongest closer to the potential target locations and diminishes as distance increases (Munoz & Istvan, 1998). Greater restriction of potential target locations facilitates this process (Basso & Wurtz, 1998). Thus, in Experiment 1, when the remote distractor is present, its associated activity is lower generally and is also lower when the remote distractor is closer to the target and greater when further away. Because of this, the general impact of the remote distractor effect on fixation disengagement would be smaller and thus latencies would be shorter in Experiment 1 than in Experiment 2. Furthermore, saccade latencies when the remote distractor was further from the target activation were less inhibited by the target and thus their impact on saccade latencies, the remote distractor effect, was greater. The same inhibitory process can be said to operate in Experiment 2, but here inhibition is less spatially specified because of increased uncertainty in target location. Due to this, any changes in the level of inhibition (and, hence, activity at remote distractor sites) due to shifts of remote distractor distance from the target are weakened, and the effect of remote distractor distance from target will also reduce. This pattern of saccade behavior is what we report here.

The results presented in this article mirror those from our previous study (McSorley & Cruickshank, 2010) in which we showed saccade accuracy improvements to a target in the presence of a close distractor as a function of a remote distractor. The experiments reported here extend that study in two ways. First, the remote distractor used by McSorley and Cruickshank (2010) was only shown in a single location, in the contralateral field to the target and close distractor, which may have been simply too far away from the target and close distractor to affect target selection processes. Using a large number of remote distractor locations shows that it does not directly impact on target selection processes, regardless of where it is shown. Second, in our earlier study, we used a gap/overlap manipulation to elicit a range of saccade latencies. As one of our aims was to examine whether the remote distractor impacted on activation at fixation, then changing when the fixation stimulus is removed relative to when the experimental stimuli appeared may have affected our results. Replicating the basic finding without this confound was vital to ensuring it was indeed the presence of the remote distractor that slowed the initiation of the saccadic response.

In conclusion, our results suggest that the involuntary increase in saccade latency induced by a remote distractor inhibits the initiation of a saccade by increasing activity at fixation, thereby interrupting fixation disengagement. This allows the target selection processes to proceed as they would ordinarily. Thus, the delay in saccade initiation induced by the presence of a remote distractor improves target localization by accessing target selection at a later, and more finely resolved, stage of development. We suggest that this may have implications in the design of any visual interface in which accuracy of visual information scanning is an important component. The results of this article suggest that sometimes adding more, potentially distracting information actually improves saccade accuracy with only a small cost in terms of the slowing of saccade initiation.

## Supplementary Material

Supplement 1
