# Supplementary material for: Inhibition of saccade initiation improves saccade accuracy: The role of local and remote visual distractors in the control of saccadic eye movements
Source: J Vis. 2021 Mar 17;21(3):17. doi: 10.1167/jov.21.3.17 (PMC7980046; doi:10.1167/jov.21.3.17)
Supplement: Supplement 1 [file jovi-21-3-17_s001.docx]

Table 1: Median saccade latencies shown for each participant by target distance, local distractor presence and remote distractor distance from fixation and remote distractor angle from the target position. A) Target at 3 degrees of visual angle from fixation with no local distractor present at the 6 degree position; B) Target at 3 degrees of visual angle from fixation with a local distractor present at the 6 degree position; C) Target at 6 degrees of visual angle from fixation with no local distractor present at the 3 degree position; D) Target at 6 degrees of visual angle from fixation with no local distractor present at the 3 degree position.

A) Target 3 degrees from center with **No Local Distractor**

| **Remote Distractor Distance** | **None** | **Center** | **3** | | | | **6** | | | | **9** | | | | **Average Non-Center** |
| --- | --- | --- | --- | --- | --- | --- | --- | --- | --- | --- | --- | --- | --- | --- | --- |
| **Remote Distractor Angle** | **None** | **Center** | **45** | **90** | **135** | **180** | **45** | **90** | **135** | **180** | **45** | **90** | **135** | **180** |  |
| **Participant 1** | 155 | 175 | 175 | 172 | 171 | 154 | 169 | 165 | 170 | 167 | 166 | 145 | 177 | 173 | 167 |
| **Participant 2** | 221 | 214 | 209 | 213 | 216 | 240 | 207 | 201 | 211 | 203 | 212 | 196 | 217 | 193 | 210 |
| **Participant 3** | 194 | 201 | 205 | 201 | 215 | 234 | 193 | 199 | 207 | 219 | 195 | 191 | 217 | 223 | 208 |
| **Participant 4** | 198 | 189 | 185 | 179 | 200 | 226 | 181 | 185 | 187 | 213 | 181 | 183 | 196 | 194 | 193 |
| **Participant 5** | 214 | 227 | 221 | 227 | 225 | 229 | 223 | 215 | 223 | 233 | 219 | 227 | 223 | 223 | 224 |
| **Participant 6** | 186 | 183 | 188 | 185 | 195 | 207 | 189 | 187 | 191 | 229 | 179 | 186 | 177 | 187 | 192 |
| **Participant 7** | 189 | 191 | 189 | 187 | 192 | 185 | 185 | 185 | 191 | 191 | 185 | 187 | 197 | 199 | 189 |
| **Average of Medians** | 194 | 197 | 196 | 195 | 202 | 211 | 192 | 191 | 197 | 208 | 191 | 188 | 201 | 199 | 198 |
| **Within Ss Standard Error** | 3.3 | 2.2 | 1.8 | 2.5 | 0.9 | 6.7 | 2.2 | 1.1 | 1.0 | 5.8 | 2.1 | 3.3 | 3.2 | 4.1 | 2.9 |

B) Target 3 degrees from center **with Local Distractor**

| **Remote Distractor Distance** | **None** | **Center** | **3** | | | | **6** | | | | **9** | | | | **Average Non-Center** |
| --- | --- | --- | --- | --- | --- | --- | --- | --- | --- | --- | --- | --- | --- | --- | --- |
| **Remote Distractor Angle** | **None** | **Center** | **45** | **90** | **135** | **180** | **45** | **90** | **135** | **180** | **45** | **90** | **135** | **180** |  |
| **Participant 1** | 143 | 181 | 182 | 176 | 179 | 157 | 173 | 176 | 173 | 176 | 171 | 177 | 187 | 179 | 176 |
| **Participant 2** | 202 | 199 | 199 | 203 | 207 | 202 | 207 | 199 | 195 | 204 | 193 | 209 | 214 | 199 | 203 |
| **Participant 3** | 189 | 196 | 203 | 196 | 213 | 237 | 195 | 200 | 204 | 235 | 184 | 200 | 202 | 223 | 208 |
| **Participant 4** | 181 | 181 | 187 | 187 | 189 | 187 | 176 | 177 | 181 | 182 | 181 | 179 | 187 | 181 | 183 |
| **Participant 5** | 212 | 221 | 219 | 215 | 216 | 224 | 217 | 215 | 214 | 215 | 217 | 223 | 214 | 227 | 218 |
| **Participant 6** | 181 | 187 | 190 | 190 | 195 | 198 | 177 | 193 | 193 | 207 | 191 | 181 | 189 | 195 | 192 |
| **Participant 7** | 181 | 183 | 185 | 191 | 187 | 195 | 181 | 185 | 189 | 186 | 179 | 187 | 181 | 179 | 185 |
| **Average of Medians** | 184 | 193 | 195 | 194 | 198 | 200 | 189 | 192 | 193 | 201 | 188 | 194 | 196 | 198 | 195 |
| **Within Ss Standard Error** | 4.0 | 2.0 | 1.5 | 2.0 | 1.2 | 5.4 | 2.4 | 1.3 | 1.4 | 4.5 | 2.9 | 2.3 | 2.8 | 3.1 | 2.6 |

C) Target 6 degrees from center with **No Local Distractor**

| **Remote Distractor Distance** | **None** | **Center** | **3** | | | | **6** | | | | **9** | | | | **Average Non-Center** |
| --- | --- | --- | --- | --- | --- | --- | --- | --- | --- | --- | --- | --- | --- | --- | --- |
| **Remote Distractor Angle** | **None** | **Center** | **45** | **90** | **135** | **180** | **45** | **90** | **135** | **180** | **45** | **90** | **135** | **180** |  |
| **Participant 1** | 183 | 174 | 211 | 203 | 199 | 211 | 185 | 189 | 193 | 178 | 193 | 201 | 177 | 189 | 194 |
| **Participant 2** | 211 | 219 | 217 | 223 | 217 | 240 | 215 | 211 | 227 | 219 | 201 | 196 | 203 | 231 | 217 |
| **Participant 3** | 187 | 189 | 193 | 190 | 194 | 207 | 184 | 191 | 185 | 197 | 183 | 189 | 189 | 191 | 191 |
| **Participant 4** | 180 | 183 | 197 | 191 | 197 | 208 | 177 | 179 | 189 | 187 | 181 | 179 | 185 | 185 | 188 |
| **Participant 5** | 213 | 221 | 234 | 229 | 234 | 234 | 213 | 227 | 221 | 227 | 211 | 215 | 228 | 225 | 225 |
| **Participant 6** | 180 | 181 | 192 | 205 | 211 | 220 | 181 | 187 | 177 | 193 | 183 | 189 | 195 | 185 | 193 |
| **Participant 7** | 180 | 183 | 189 | 185 | 187 | 183 | 185 | 183 | 183 | 181 | 179 | 181 | 177 | 191 | 184 |
| **Average of Medians** | 153 | 164 | 164 | 168 | 171 | 178 | 156 | 158 | 160 | 169 | 153 | 158 | 162 | 165 | 199 |
| **Within Ss Standard Error** | 1.2 | 2.6 | 2.5 | 1.9 | 2.4 | 3.6 | 1.8 | 1.5 | 2.9 | 2.5 | 2.1 | 3.4 | 2.9 | 2.7 | 2.5 |

D) Target 6 degrees from center with **Local Distractor**

| **Remote Distractor Distance** | **None** | **Center** | **3** | | | | **6** | | | | **9** | | | | **Average Non-Center** |
| --- | --- | --- | --- | --- | --- | --- | --- | --- | --- | --- | --- | --- | --- | --- | --- |
| **Remote Distractor Angle** | **None** | **Center** | **45** | **90** | **135** | **180** | **45** | **90** | **135** | **180** | **45** | **90** | **135** | **180** |  |
| **Participant 1** | 177 | 177 | 185 | 178 | 183 | 183 | 183 | 183 | 181 | 179 | 167 | 173 | 189 | 173 | 180 |
| **Participant 2** | 189 | 212 | 205 | 202 | 209 | 210 | 193 | 193 | 201 | 199 | 193 | 205 | 202 | 200 | 201 |
| **Participant 3** | 191 | 193 | 199 | 197 | 209 | 227 | 191 | 188 | 190 | 213 | 190 | 187 | 205 | 204 | 200 |
| **Participant 4** | 177 | 185 | 185 | 185 | 193 | 200 | 185 | 185 | 186 | 193 | 184 | 183 | 189 | 176 | 187 |
| **Participant 5** | 217 | 213 | 219 | 228 | 226 | 218 | 219 | 215 | 215 | 225 | 221 | 217 | 216 | 231 | 221 |
| **Participant 6** | 179 | 187 | 186 | 193 | 203 | 216 | 177 | 177 | 192 | 191 | 179 | 188 | 195 | 208 | 192 |
| **Participant 7** | 182 | 186 | 185 | 182 | 183 | 185 | 184 | 179 | 186 | 185 | 185 | 182 | 179 | 187 | 184 |
| **Average of Medians** | 187 | 193 | 195 | 195 | 201 | 206 | 190 | 189 | 193 | 198 | 188 | 191 | 196 | 197 | 195 |
| **Within Ss Standard Error** | 1.7 | 2.3 | 1.3 | 1.3 | 1.6 | 4.4 | 2.3 | 2.2 | 1.6 | 2.1 | 2.2 | 1.8 | 2.0 | 3.6 | 2.2 |

Table 2: Average saccade landing positions (amplitudes are shown) in degrees of visual angle. Four tables detail average data for each participant by target distance, local distractor presence and remote distractor distance from fixation and remote distractor angle from the target position. A) Target at 3 degrees of visual angle from fixation with no local distractor present at the 6 degree position; B) Target at 3 degrees of visual angle from fixation with a local distractor present at the 6 degree position; C) Target at 6 degrees of visual angle from fixation with no local distractor present at the 3 degree position; D) Target at 6 degrees of visual angle from fixation with no local distractor present at the 3 degree position.

A) Target 3 degrees from center with **No Local Distractor**

| **Remote Distractor Distance** | **None** | **Center** | **3** | | | | **6** | | | | **9** | | | | **Average Non-Center** |
| --- | --- | --- | --- | --- | --- | --- | --- | --- | --- | --- | --- | --- | --- | --- | --- |
| **Remote Distractor Angle** | **None** | **Center** | **45** | **90** | **135** | **180** | **45** | **90** | **135** | **180** | **45** | **90** | **135** | **180** |  |
| **Participant 1** | 2.5 | 2.5 | 2.5 | 2.6 | 2.6 | 2.5 | 2.6 | 2.5 | 2.6 | 2.5 | 2.5 | 2.3 | 2.5 | 2.5 | 2.5 |
| **Participant 2** | 2.3 | 2.3 | 2.2 | 2.2 | 2.3 | 2.3 | 2.2 | 2.3 | 2.2 | 2.3 | 2.3 | 2.3 | 2.4 | 2.4 | 2.3 |
| **Participant 3** | 3.0 | 2.9 | 2.8 | 2.9 | 2.9 | 3.0 | 2.8 | 2.9 | 2.9 | 2.9 | 2.6 | 2.9 | 2.9 | 3.0 | 2.9 |
| **Participant 4** | 2.8 | 2.7 | 2.7 | 2.7 | 2.8 | 2.9 | 2.7 | 2.8 | 2.8 | 2.9 | 2.5 | 2.8 | 2.9 | 2.9 | 2.8 |
| **Participant 5** | 2.6 | 2.7 | 2.6 | 2.5 | 2.5 | 2.6 | 2.6 | 2.5 | 2.6 | 2.7 | 2.3 | 2.1 | 2.3 | 2.5 | 2.5 |
| **Participant 6** | 2.7 | 2.9 | 2.6 | 2.8 | 2.6 | 2.6 | 2.6 | 2.7 | 2.8 | 2.9 | 2.6 | 2.7 | 2.5 | 2.7 | 2.7 |
| **Participant 7** | 2.7 | 2.8 | 2.4 | 2.6 | 2.5 | 2.5 | 2.6 | 2.7 | 2.7 | 2.6 | 2.3 | 2.3 | 2.4 | 2.6 | 2.5 |
| **Average of Medians** | 2.7 | 2.7 | 2.6 | 2.6 | 2.6 | 2.6 | 2.6 | 2.6 | 2.7 | 2.7 | 2.4 | 2.5 | 2.5 | 2.6 | 2.6 |
| **Within Ss Standard Error** | 2.7 | 2.7 | 2.6 | 2.6 | 2.6 | 2.6 | 2.6 | 2.6 | 2.7 | 2.7 | 2.4 | 2.5 | 2.5 | 2.6 | 2.6 |

B) Target 3 degrees from center **with Local Distractor**

| **Remote Distractor Distance** | **None** | **Center** | **3** | | | | **6** | | | | **9** | | | | **Average Non-Center** |
| --- | --- | --- | --- | --- | --- | --- | --- | --- | --- | --- | --- | --- | --- | --- | --- |
| **Remote Distractor Angle** | **None** | **Center** | **45** | **90** | **135** | **180** | **45** | **90** | **135** | **180** | **45** | **90** | **135** | **180** |  |
| **Participant 1** | 2.9 | 2.9 | 2.9 | 2.8 | 3.0 | 2.6 | 3.0 | 2.9 | 3.0 | 2.7 | 2.8 | 2.8 | 2.8 | 3.0 | 2.8 |
| **Participant 2** | 2.6 | 2.6 | 2.4 | 2.5 | 2.5 | 2.4 | 2.6 | 2.4 | 2.5 | 2.6 | 2.8 | 2.6 | 2.6 | 2.4 | 2.5 |
| **Participant 3** | 3.4 | 3.4 | 3.3 | 3.1 | 3.3 | 3.4 | 3.4 | 3.3 | 3.4 | 3.4 | 3.2 | 3.2 | 3.1 | 3.4 | 3.3 |
| **Participant 4** | 3.1 | 3.2 | 3.0 | 3.1 | 3.0 | 3.2 | 3.1 | 3.2 | 3.2 | 2.9 | 3.1 | 3.1 | 3.1 | 3.1 | 3.1 |
| **Participant 5** | 2.7 | 3.2 | 2.7 | 2.8 | 2.7 | 3.0 | 3.0 | 2.9 | 2.9 | 2.6 | 2.5 | 2.3 | 2.4 | 2.8 | 2.7 |
| **Participant 6** | 3.5 | 3.4 | 3.2 | 3.0 | 2.9 | 2.8 | 3.7 | 3.1 | 3.2 | 3.0 | 3.1 | 3.1 | 3.1 | 3.3 | 3.1 |
| **Participant 7** | 3.0 | 3.5 | 3.2 | 2.9 | 3.2 | 2.9 | 3.6 | 3.2 | 2.9 | 2.8 | 3.0 | 2.7 | 2.7 | 3.1 | 3.0 |
| **Average of Medians** | 3.0 | 3.2 | 3.0 | 2.9 | 2.9 | 2.9 | 3.2 | 3.0 | 3.0 | 2.9 | 2.9 | 2.8 | 2.8 | 3.0 | 2.9 |
| **Within Ss Standard Error** | 3.0 | 3.2 | 3.0 | 2.9 | 2.9 | 2.9 | 3.2 | 3.0 | 3.0 | 2.9 | 2.9 | 2.8 | 2.8 | 3.0 | 3.0 |

C) Target 6 degrees from center with **No Local Distractor**

| **Remote Distractor Distance** | **None** | **Center** | **3** | | | | **6** | | | | **9** | | | | **Average Non-Center** |
| --- | --- | --- | --- | --- | --- | --- | --- | --- | --- | --- | --- | --- | --- | --- | --- |
| **Remote Distractor Angle** | **None** | **Center** | **45** | **90** | **135** | **180** | **45** | **90** | **135** | **180** | **45** | **90** | **135** | **180** |  |
| **Participant 1** | 4.7 | 4.9 | 4.7 | 5.1 | 5.0 | 5.0 | 5.0 | 4.8 | 5.0 | 5.0 | 5.2 | 5.0 | 5.1 | 5.0 | 5.0 |
| **Participant 2** | 4.6 | 4.7 | 4.1 | 4.7 | 4.7 | 4.8 | 4.8 | 4.8 | 4.7 | 4.8 | 4.9 | 4.8 | 4.8 | 4.8 | 4.7 |
| **Participant 3** | 6.1 | 6.5 | 5.9 | 6.3 | 6.4 | 6.7 | 6.1 | 6.2 | 6.4 | 6.4 | 5.8 | 6.1 | 6.2 | 6.1 | 6.2 |
| **Participant 4** | 6.0 | 5.9 | 5.4 | 6.0 | 6.1 | 6.2 | 5.7 | 6.0 | 6.0 | 6.3 | 5.6 | 6.0 | 5.9 | 6.2 | 5.9 |
| **Participant 5** | 5.5 | 5.6 | 5.0 | 5.3 | 5.5 | 5.3 | 5.3 | 5.4 | 5.4 | 5.3 | 5.2 | 5.1 | 5.0 | 5.3 | 5.3 |
| **Participant 6** | 5.2 | 5.6 | 4.8 | 5.3 | 5.4 | 5.5 | 5.0 | 5.1 | 5.5 | 5.4 | 4.9 | 4.9 | 5.1 | 5.3 | 5.2 |
| **Participant 7** | 5.1 | 4.9 | 4.6 | 4.8 | 5.0 | 5.0 | 5.0 | 4.9 | 4.8 | 4.9 | 4.8 | 4.4 | 4.9 | 5.0 | 4.8 |
| **Average of Medians** | 5.3 | 5.4 | 4.9 | 5.4 | 5.4 | 5.5 | 5.3 | 5.3 | 5.4 | 5.5 | 5.2 | 5.2 | 5.3 | 5.4 | 5.3 |
| **Within Ss Standard Error** | 5.3 | 5.4 | 4.9 | 5.4 | 5.4 | 5.5 | 5.3 | 5.3 | 5.4 | 5.5 | 5.2 | 5.2 | 5.3 | 5.4 | 5.3 |

D) Target 6 degrees from center with **Local Distractor**

| **Remote Distractor Distance** | **None** | **Center** | **3** | | | | **6** | | | | **9** | | | | **Average Non-Center** |
| --- | --- | --- | --- | --- | --- | --- | --- | --- | --- | --- | --- | --- | --- | --- | --- |
| **Remote Distractor Angle** | **None** | **Center** | **45** | **90** | **135** | **180** | **45** | **90** | **135** | **180** | **45** | **90** | **135** | **180** |  |
| **Participant 1** | 2.8 | 3.1 | 3.3 | 3.1 | 3.2 | 2.8 | 3.2 | 2.8 | 3.0 | 3.0 | 3.2 | 3.1 | 3.3 | 2.8 | 3.1 |
| **Participant 2** | 3.5 | 3.5 | 3.3 | 3.4 | 3.2 | 3.3 | 3.3 | 3.3 | 3.5 | 3.2 | 3.6 | 3.3 | 3.4 | 3.6 | 3.4 |
| **Participant 3** | 4.8 | 5.2 | 5.0 | 4.8 | 5.2 | 5.6 | 4.7 | 4.7 | 4.9 | 4.7 | 4.7 | 4.6 | 4.6 | 5.0 | 4.9 |
| **Participant 4** | 4.4 | 4.8 | 4.6 | 4.8 | 4.8 | 5.1 | 4.6 | 4.6 | 4.8 | 4.6 | 4.4 | 4.6 | 4.4 | 4.5 | 4.6 |
| **Participant 5** | 4.3 | 4.8 | 4.4 | 4.4 | 4.4 | 4.3 | 4.1 | 4.3 | 4.2 | 4.7 | 4.5 | 4.2 | 3.8 | 4.7 | 4.3 |
| **Participant 6** | 3.7 | 4.1 | 3.7 | 3.5 | 3.4 | 3.4 | 3.7 | 3.6 | 3.7 | 3.4 | 3.3 | 3.5 | 3.1 | 3.7 | 3.5 |
| **Participant 7** | 3.9 | 4.5 | 3.9 | 4.0 | 3.9 | 4.2 | 4.1 | 4.1 | 4.0 | 3.8 | 3.7 | 3.5 | 3.9 | 3.7 | 3.9 |
| **Average of Medians** | 3.9 | 4.3 | 4.0 | 4.0 | 4.0 | 4.1 | 4.0 | 3.9 | 4.0 | 3.9 | 3.9 | 3.8 | 3.8 | 4.0 | 4.0 |
| **Within Ss Standard Error** | 3.9 | 4.3 | 4.0 | 4.0 | 4.0 | 4.1 | 4.0 | 3.9 | 4.0 | 3.9 | 3.9 | 3.8 | 3.8 | 4.0 | 4.0 |

Table 3: Median saccade latencies shown for each participant by target distance, local distractor presence and remote distractor distance from fixation and remote distractor angle from the target position. A) Target at 10 degrees of visual angle from fixation with no local distractor present; B) Target at 10 degrees of visual angle from fixation with a local distractor present; C) Target at 20 degrees of visual angle from fixation with no local distractor present; D) Target at 20 degrees of visual angle from fixation with no local distractor present.

A) Target 10 degrees from center with **No Local Distractor**

| **Remote Distractor Distance** | **None** | **Center** | **3** | | | | | **6** | | | | | **Average Non-Center** |
| --- | --- | --- | --- | --- | --- | --- | --- | --- | --- | --- | --- | --- | --- |
| **Remote Distractor Angle** | **None** | **Center** | **50** | **70** | **110** | **130** | **170** | **50** | **70** | **110** | **130** | **170** |  |
| **Participant 1** | 171 | 211 | 191 | 198 | 193 | 181 | 187 | 185 | 180 | 190 | 185 | 193 | 188 |
| **Participant 2** | 153 | 171 | 211 | 181 | 188 | 163 | 159 | 171 | 138 | 160 | 139 | 166 | 168 |
| **Participant 3** | 161 | 216 | 181 | 199 | 185 | 185 | 210 | 162 | 174 | 167 | 210 | 175 | 185 |
| **Participant 4** | 138 | 212 | 173 | 193 | 183 | 169 | 180 | 160 | 143 | 188 | 159 | 171 | 172 |
| **Participant 5** | 161 | 179 | 179 | 213 | 178 | 166 | 178 | 162 | 142 | 161 | 151 | 193 | 172 |
| **Participant 6** | 155 | 288 | 230 | 226 | 175 | 195 | 184 | 191 | 175 | 181 | 184 | 184 | 192 |
| **Participant 7** | 133 | 174 | 164 | 178 | 150 | 151 | 175 | 174 | 170 | 149 | 165 | 155 | 163 |
| **Average of Medians** | 153 | 207 | 190 | 198 | 179 | 173 | 182 | 172 | 160 | 171 | 170 | 177 | 178 |
| **Within Ss Standard Error** | 4.3 | 11.2 | 7.1 | 4.2 | 5.4 | 1.3 | 4.9 | 4.3 | 5.4 | 4.5 | 6.6 | 4.3 | 5.3 |

B) Target 10 degrees from center with **Local Distractor**

| **Remote Distractor Distance** | **None** | **Center** | **3** | | | | | **6** | | | | | **Average Non-Center** |
| --- | --- | --- | --- | --- | --- | --- | --- | --- | --- | --- | --- | --- | --- |
| **Remote Distractor Angle** | **None** | **Center** | **50** | **70** | **110** | **130** | **170** | **50** | **70** | **110** | **130** | **170** |  |
| **Participant 1** | 169 | 211 | 181 | 187 | 185 | 193 | 179 | 175 | 164 | 185 | 175 | 186 | 181 |
| **Participant 2** | 148 | 218 | 179 | 181 | 179 | 179 | 174 | 149 | 162 | 158 | 154 | 177 | 169 |
| **Participant 3** | 160 | 211 | 169 | 178 | 175 | 171 | 189 | 168 | 145 | 203 | 164 | 185 | 175 |
| **Participant 4** | 151 | 200 | 144 | 168 | 165 | 152 | 188 | 152 | 150 | 154 | 151 | 141 | 156 |
| **Participant 5** | 164 | 210 | 209 | 212 | 180 | 182 | 169 | 163 | 172 | 181 | 175 | 166 | 181 |
| **Participant 6** | 151 | 235 | 194 | 210 | 158 | 183 | 178 | 160 | 153 | 156 | 173 | 167 | 173 |
| **Participant 7** | 149 | 178 | 168 | 165 | 167 | 158 | 151 | 150 | 160 | 167 | 153 | 157 | 159 |
| **Average of Medians** | 156 | 209 | 178 | 186 | 173 | 174 | 175 | 159 | 158 | 172 | 163 | 168 | 173 |
| **Within Ss Standard Error** | 2.3 | 5.0 | 5.4 | 4.9 | 3.4 | 2.7 | 5.3 | 2.4 | 4.0 | 5.7 | 1.7 | 4.0 | 3.9 |

C) Target 20 degrees from center with **No Local Distractor**

| **Remote Distractor Distance** | **None** | **Center** | **3** | | | | | **6** | | | | | **Average non-Center** |
| --- | --- | --- | --- | --- | --- | --- | --- | --- | --- | --- | --- | --- | --- |
| **Remote Distractor Angle** | **None** | **Center** | **50** | **70** | **110** | **130** | **170** | **50** | **70** | **110** | **130** | **170** |  |
| **Participant 1** | 168 | 220 | 197 | 179 | 199 | 189 | 207 | 191 | 185 | 182 | 181 | 198 | 191 |
| **Participant 2** | 167 | 207 | 236 | 198 | 182 | 158 | 188 | 173 | 159 | 179 | 171 | 165 | 181 |
| **Participant 3** | 190 | 255 | 206 | 217 | 195 | 188 | 230 | 203 | 186 | 196 | 186 | 213 | 202 |
| **Participant 4** | 140 | 222 | 183 | 175 | 204 | 179 | 197 | 173 | 159 | 188 | 173 | 178 | 181 |
| **Participant 5** | 148 | 186 | 206 | 182 | 189 | 163 | 189 | 178 | 164 | 206 | 176 | 185 | 184 |
| **Participant 6** | 155 | 257 | 270 | 244 | 192 | 190 | 215 | 178 | 162 | 161 | 185 | 210 | 201 |
| **Participant 7** | 141 | 215 | 186 | 180 | 172 | 159 | 164 | 160 | 158 | 156 | 157 | 170 | 166 |
| **Average of Medians** | 158 | 223 | 212 | 196 | 191 | 175 | 199 | 180 | 168 | 181 | 176 | 188 | 187 |
| **Within Ss Standard Error** | 4.1 | 5.1 | 10.0 | 7.0 | 4.0 | 3.2 | 3.6 | 2.9 | 4.1 | 7.2 | 1.3 | 3.5 | 4.7 |

D) Target 20 degrees from center with **Local Distractor**

| **Remote Distractor Distance** | **None** | **Center** | **3** | | | | | **6** | | | | | **Average Non-Center** |
| --- | --- | --- | --- | --- | --- | --- | --- | --- | --- | --- | --- | --- | --- |
| **Remote Distractor Angle** | **None** | **Center** | **50** | **70** | **110** | **130** | **170** | **50** | **70** | **110** | **130** | **170** |  |
| **Participant 1** | 180 | 228 | 180 | 192 | 202 | 190 | 199 | 184 | 186 | 208 | 190 | 188 | 192 |
| **Participant 2** | 166 | 220 | 218 | 176 | 205 | 165 | 194 | 176 | 172 | 164 | 170 | 184 | 182 |
| **Participant 3** | 160 | 208 | 215 | 218 | 192 | 188 | 207 | 182 | 164 | 192 | 197 | 204 | 196 |
| **Participant 4** | 170 | 231 | 174 | 188 | 207 | 186 | 187 | 180 | 169 | 177 | 175 | 176 | 182 |
| **Participant 5** | 142 | 183 | 214 | 177 | 181 | 174 | 166 | 155 | 170 | 177 | 202 | 186 | 180 |
| **Participant 6** | 160 | 261 | 213 | 218 | 180 | 191 | 193 | 180 | 195 | 166 | 177 | 179 | 189 |
| **Participant 7** | 177 | 188 | 160 | 165 | 163 | 166 | 182 | 166 | 159 | 174 | 166 | 157 | 166 |
| **Average of Medians** | 165 | 217 | 196 | 191 | 190 | 180 | 189 | 175 | 173 | 180 | 182 | 182 | 185 |
| **Within Ss Standard Error** | 4.8 | 5.9 | 7.7 | 5.1 | 5.0 | 2.8 | 3.7 | 3.2 | 4.3 | 5.2 | 4.4 | 2.6 | 4.6 |

Table 4: Average saccade landing positions (amplitudes are shown) in degrees of visual angle. Four tables detail average data for each participant by target distance, local distractor presence and remote distractor distance from fixation and remote distractor angle from the target position. A) Target at 10 degrees of visual angle from fixation with no local distractor present; B) Target at 10 degrees of visual angle from fixation with a local distractor present; C) Target at 20 degrees of visual angle from fixation with no local distractor present; D) Target at 20 degrees of visual angle from fixation with no local distractor present.

A) Target 10 degrees from center with **No Local Distractor**

| **Remote Distractor Distance** | **None** | **Center** | **3** | | | | | **6** | | | | | **Average Non-Center** |
| --- | --- | --- | --- | --- | --- | --- | --- | --- | --- | --- | --- | --- | --- |
| **Remote Distractor Angle** | **None** | **Center** | **50** | **70** | **110** | **130** | **170** | **50** | **70** | **110** | **130** | **170** |  |
| **Participant 1** | 10.1 | 10.0 | 7.0 | 14.3 | 6.7 | 13.4 | 10.6 | 6.5 | 13.4 | 8.2 | 13.5 | 10.6 | 10.1 |
| **Participant 2** | 7.6 | 8.6 | 22.1 | 6.3 | 11.3 | 7.2 | 8.6 | 9.8 | 7.8 | 11.9 | 7.3 | 11.1 | 7.6 |
| **Participant 3** | 8.8 | 6.0 | 8.3 | 5.1 | 10.1 | 7.3 | 7.5 | 5.7 | 7.4 | 8.1 | 5.2 | 9.5 | 8.8 |
| **Participant 4** | 10.1 | 9.9 | 14.1 | 8.6 | 11.1 | 7.3 | 8.0 | 12.4 | 7.3 | 9.6 | 7.1 | 9.4 | 10.1 |
| **Participant 5** | 4.0 | 8.4 | 13.2 | 1.3 | 16.2 | 5.9 | 8.1 | 10.9 | 4.8 | 11.1 | 10.1 | 5.5 | 4.0 |
| **Participant 6** | 8.2 | 8.9 | 20.4 | 13.8 | 13.5 | 4.5 | 9.4 | 14.7 | 3.1 | 12.1 | 5.0 | 9.0 | 8.2 |
| **Participant 7** | 8.5 | 8.1 | 8.0 | 9.3 | 7.2 | 9.9 | 9.1 | 6.6 | 10.1 | 6.7 | 9.0 | 8.0 | 8.5 |
| **Average of Medians** | 8.2 | 8.6 | 13.3 | 8.4 | 10.9 | 7.9 | 8.8 | 9.5 | 7.7 | 9.7 | 8.2 | 9.0 | 8.2 |
| **Within Ss Standard Error** | 0.8 | 0.3 | 2.1 | 1.5 | 1.4 | 1.1 | 0.3 | 1.1 | 1.3 | 0.7 | 1.1 | 0.6 | 0.8 |

B) Target 10 degrees from center **with Local Distractor**

| **Remote Distractor Distance** | **None** | **Center** | **3** | | | | | **6** | | | | | **Average Non-Center** |
| --- | --- | --- | --- | --- | --- | --- | --- | --- | --- | --- | --- | --- | --- |
| **Remote Distractor Angle** | **None** | **Center** | **50** | **70** | **110** | **130** | **170** | **50** | **70** | **110** | **130** | **170** |  |
| **Participant 1** | 5.1 | 4.9 | 1.7 | 8.9 | 2.2 | 9.1 | 5.0 | 3.0 | 10.6 | 0.1 | 8.5 | 3.8 | 10.4 |
| **Participant 2** | 0.5 | 7.7 | 4.0 | 7.3 | 1.9 | -0.8 | 3.7 | 3.3 | 2.8 | 5.0 | 1.2 | 5.4 | 10.0 |
| **Participant 3** | -0.7 | 2.2 | 4.7 | 2.7 | 1.0 | 3.9 | 2.3 | -0.3 | -0.4 | 0.3 | 0.5 | 3.4 | 7.4 |
| **Participant 4** | 0.9 | 7.5 | 6.0 | 2.5 | 5.6 | 2.0 | 3.4 | 5.6 | 2.0 | 3.2 | 1.4 | 1.1 | 9.6 |
| **Participant 5** | 5.9 | 6.7 | 8.0 | 5.7 | 4.8 | 1.1 | 5.8 | 3.2 | 0.2 | 4.6 | 1.0 | 5.5 | 8.3 |
| **Participant 6** | 1.8 | 3.7 | 7.8 | 2.5 | 6.5 | -2.1 | 1.2 | 8.1 | -2.8 | 6.1 | -2.2 | 1.6 | 10.2 |
| **Participant 7** | 3.0 | 3.9 | -2.1 | 1.9 | 4.5 | 0.8 | 4.6 | 2.4 | 2.3 | 3.5 | 2.1 | 0.1 | 8.4 |
| **Average of Medians** | 2.3 | 5.2 | 4.3 | 4.5 | 3.8 | 2.0 | 3.7 | 3.6 | 2.1 | 3.3 | 1.8 | 3.0 | 9.2 |
| **Within Ss Standard Error** | 0.7 | 0.7 | 1.4 | 0.7 | 0.9 | 1.2 | 0.5 | 1.0 | 1.3 | 1.0 | 1.0 | 0.7 | 1.0 |

C) Target 20 degrees from center with **No Local Distractor**

| **Remote Distractor Distance** | **None** | **Center** | **3** | | | | | **6** | | | | | **Average Non-Center** |
| --- | --- | --- | --- | --- | --- | --- | --- | --- | --- | --- | --- | --- | --- |
| **Remote Distractor Angle** | **None** | **Center** | **50** | **70** | **110** | **130** | **170** | **50** | **70** | **110** | **130** | **170** |  |
| **Participant 1** | 15.5 | 21.8 | 19.3 | 26.9 | 15.7 | 25.4 | 20.4 | 19.3 | 26.1 | 17.0 | 25.4 | 20.6 | 10.4 |
| **Participant 2** | 13.3 | 20.0 | 31.4 | 26.9 | 22.3 | 16.1 | 18.9 | 23.2 | 18.0 | 25.6 | 20.0 | 20.3 | 10.0 |
| **Participant 3** | 13.0 | 18.0 | 19.1 | 15.6 | 20.0 | 14.8 | 22.4 | 18.2 | 16.1 | 17.5 | 16.2 | 19.3 | 7.4 |
| **Participant 4** | 15.8 | 19.3 | 23.7 | 18.9 | 21.1 | 16.0 | 18.5 | 22.2 | 19.1 | 20.1 | 16.8 | 21.0 | 9.6 |
| **Participant 5** | 14.3 | 22.3 | 20.7 | 21.1 | 19.5 | 20.6 | 20.8 | 24.1 | 22.0 | 17.7 | 20.9 | 20.4 | 8.3 |
| **Participant 6** | 14.7 | 20.0 | 34.2 | 20.0 | 27.9 | 18.3 | 21.4 | 26.7 | 12.4 | 24.8 | 15.8 | 22.4 | 10.2 |
| **Participant 7** | 12.0 | 19.9 | 17.0 | 20.7 | 16.2 | 21.1 | 18.4 | 15.4 | 16.6 | 17.6 | 21.5 | 17.4 | 8.4 |
| **Average of Medians** | 14.1 | 20.2 | 23.6 | 21.4 | 20.4 | 18.9 | 20.1 | 21.3 | 18.6 | 20.0 | 19.5 | 20.2 | 9.2 |
| **Within Ss Standard Error** | 0.6 | 0.5 | 2.1 | 1.2 | 1.4 | 1.4 | 0.9 | 1.1 | 1.7 | 1.1 | 1.3 | 0.4 | 1.0 |

D) Target 20 degrees from center with **Local Distractor**

| **Remote Distractor Distance** | **None** | **Center** | **3** | | | | | **6** | | | | | **Average Non-Center** |
| --- | --- | --- | --- | --- | --- | --- | --- | --- | --- | --- | --- | --- | --- |
| **Remote Distractor Angle** | **None** | **Center** | **50** | **70** | **110** | **130** | **170** | **50** | **70** | **110** | **130** | **170** |  |
| **Participant 1** | 11.7 | 19.2 | 11.0 | 19.9 | 9.3 | 22.7 | 14.6 | 12.3 | 12.4 | 15.3 | 19.1 | 18.2 | 10.4 |
| **Participant 2** | 16.9 | 17.9 | 12.5 | 10.2 | 7.6 | 10.4 | 0.6 | 19.2 | 5.3 | 9.2 | 18.4 | 13.4 | 10.0 |
| **Participant 3** | 3.2 | 8.7 | 6.5 | 6.7 | 8.4 | 15.3 | 7.6 | 4.3 | 15.2 | 7.8 | 10.0 | 7.9 | 7.4 |
| **Participant 4** | 9.4 | 16.9 | 12.8 | 13.8 | 11.4 | 9.8 | 14.1 | 19.6 | 8.2 | 10.5 | 8.9 | 16.0 | 9.6 |
| **Participant 5** | 5.7 | 7.2 | 14.3 | 10.0 | 13.6 | 8.8 | 13.8 | 7.3 | 10.6 | 12.4 | 13.7 | 13.7 | 8.3 |
| **Participant 6** | 2.9 | 16.3 | 18.1 | 14.9 | 13.3 | 6.1 | 16.4 | 9.5 | 8.8 | 17.8 | 2.3 | 10.0 | 10.2 |
| **Participant 7** | 5.6 | 19.4 | 8.5 | 13.6 | 5.9 | 7.4 | 9.0 | 9.9 | 10.3 | 3.5 | 1.4 | 11.0 | 8.4 |
| **Average of Medians** | 7.9 | 15.1 | 12.0 | 12.7 | 9.9 | 11.5 | 10.9 | 11.7 | 10.1 | 10.9 | 10.5 | 12.9 | 9.2 |
| **Within Ss Standard Error** | 1.6 | 1.7 | 1.4 | 1.1 | 1.2 | 1.8 | 2.0 | 1.9 | 1.7 | 1.4 | 2.2 | 0.7 | 1.0 |
